# β1,4-galactosyltransferase III drives retinoblastoma invasion via activation of integrin-FAK axis

**DOI:** 10.1038/s41419-026-08620-5

**Published:** 2026-03-23

**Authors:** Junjie Tang, Jinmiao Li, Meng Wang, Yaoming Liu, Hetian Sun, Zhihui Zhang, Yang Gao, Chao Cheng, Shuxia Chen, Ping Zhang, Siming Ai, Shicai Su, Youjin Hu, Rong Lu

**Affiliations:** State Key Laboratory of Ophthalmology, Zhongshan Ophthalmic Center, Sun Yat-sen University, Guangzhou, China

**Keywords:** Eye cancer, Paediatric cancer

## Abstract

Retinoblastoma (RB) is the most common primary intraocular malignancy in children, and its extraocular extension is closely linked to poor prognosis. However, the molecular drivers underlying local invasion remain incompletely defined. Here, we identify β‑1,4‑galactosyltransferase III (B4GALT3) as a glycosyltransferase selectively upregulated in highly proliferative MKI67⁺ RB subpopulations. B4GALT3 promotes RB cell proliferation, fibronectin adhesion, and invasion by enhancing β1-integrin glycosylation, thereby activating FAK signaling and inducing MMP2 expression to disrupt retinal epithelial barriers. Genetic modulation of B4GALT3 significantly altered both tumor burden and invasive behavior in orthotopic xenograft models. Structure-based virtual screening identified myricoside as a B4GALT3 inhibitor, which suppressed RB malignancy in vitro and in vivo. Overall, our findings uncover a B4GALT3–integrin–FAK axis as a key regulator of RB progression and highlight B4GALT3 inhibition as a promising therapeutic strategy for advanced RB.

## Introduction

Retinoblastoma (RB) is the most common primary intraocular malignancy in children, with approximately 9000 new cases diagnosed annually worldwide [1, 2]. Its potential for extraocular extension—marked by optic nerve invasion beyond the lamina cribrosa or scleral penetration—enables metastasis to the central nervous system, bones, or lymph nodes, correlating with a significant drop in cumulative survival from 100% in early-stage disease to 45% [3]. While recent eye-preserving therapies have significantly improved overall survival and globe salvage rates for intraocular RB, patients with advanced-stage disease and clinical high-risk features—such as secondary glaucoma, anterior chamber invasion—remain at high risk of extraocular progression [4, 5]. Therefore, identifying specific targets and strategies to halt RB extraocular invasion is crucial for improving the efficacy and safety of eye-preserving treatments.

Metabolic reprogramming induces spatiotemporal heterogeneity in tumor cells and confers invasive potential, enabling secretion of enzymes/cytokines that remodel the extracellular matrix (ECM) to promote local invasion [6–8]. During progression, RB cells invade the glycosaminoglycan-rich vitreous and penetrate the collagen/elastin-dominated sclera [9–11]. These processes likely involve dynamic cell-ECM interactions that facilitate overcoming these distinct barriers. β-1,4-galactosyltransferase III (B4GALT3), a member of the B4GALT family, catalyzes galactose transfer to GlcNAc-terminated glycans, forming poly-N-acetyllactosamine chains critical for glycoprotein and glycolipid biosynthesis [12, 13]. Emerging evidence links B4GALT3 to oncogenesis, with roles in promoting proliferation, invasion, and metastasis across neuroblastoma, cervical cancer, and colorectal cancer via β1-integrin activation and ECM remodeling [14–16]. However, its specific function in RB invasion remains unclear.

In this study, we show that B4GALT3 is highly upregulated in RB, particularly in aggressive MKI67⁺ photoreceptor-decreased (PhrD) subpopulations across intraocular and extraocular stages. Functionally, B4GALT3 promotes RB cell proliferation, adhesion, and invasion by enhancing β1-integrin glycosylation, thereby activating the FAK signaling pathway and inducing MMP2-mediated disruption of retinal epithelial barriers. These effects are validated in vivo, where B4GALT3 overexpression accelerates tumor growth and invasiveness, and knockdown attenuates progression. Notably, we identify myricoside as a B4GALT3 inhibitor that suppresses RB malignancy both in vitro and in orthotopic xenograft models, supporting B4GALT3 as a promising therapeutic target for RB.

## Methods

### Ethical approval and consent to participate

All animal experiments adhered to the ARVO Statement for the Use of Animals in Ophthalmic and Vision Research, with protocols approved by the Institutional Animal Care and Use Committee (IACUC) of Zhongshan Ophthalmic Center (Z2024096). For human subjects, written informed consent was obtained from family members of patients, and the study was approved by the Ethics Review Board of Zhongshan Ophthalmic Center (Guangzhou, China; 2023KYPJ308). All procedures conformed to the guidelines of the Declaration of Helsinki. Human retinoblastoma (RB) tissue sections were acquired from the Department of Pathology at Zhongshan Ophthalmic Center.

### Cell lines and reagents

The cell lines utilized in this research included retinoblastoma cell lines WERI-Rb1 (HTB-169, American Type Culture Collection [ATCC]) and Y79 (HTB-18, ATCC), human embryonic kidney cell line HEK293T (CRL-3216, ATCC), and human retinal pigment epithelial cell line ARPE-19 (CRL-2302, ATCC). WERI-Rb1 and Y79 cells, along with their derivative lines, were cultured in RPMI 1640 medium (Corning, USA); ARPE-19 and its derivatives were maintained in Dulbecco’s Modified Eagle Medium/Nutrient Mixture F-12 (DMEM/F-12, Gibco, Thermo Fisher Scientific, USA); HEK293T were cultured in Dulbecco’s Modified Eagle Medium (DMEM, Gibco, Thermo Fisher Scientific, USA). All media were supplemented with 10% fetal bovine serum (Gibco, Thermo Fisher Scientific, USA) and 1% penicillin-streptomycin (Invitrogen, USA). Cells were incubated at 37 °C in a humidified atmosphere containing 5% CO₂ and routinely screened for mycoplasma contamination using Hoechst staining. Compounds purchased from TargetMol (USA) were dissolved in dimethyl sulfoxide (DMSO) to generate 10 mM stock solutions, aliquoted, and stored at −20 °C; these included Salvianolic acid B (T2727), Calceolarioside B (T3899), Plantainoside D (T5796), Raffinose (T8144), Rhosin hydrochloride (T16745), Myricoside (TN7085), Clovamide (T26288), Isolugrandoside (TN7091), Hamamelitannin (TN1722), Eriodictyol-7-O-glucoside (TN1621), Regaloside C (TN6490), Diquafosol tetrasodium (T7423), NADP disodium salt (T22446), Isomaltose (T0581), and Isoforsythiaside (T3S1088). Additionally, Ilomastat (GM6001, S7157, Selleck Chemicals, USA) and PF-573228 (S2013, Selleck Chemicals, USA) were prepared using the same protocol.

### Single-cell sequencing data analysis

For single-cell metabolic activity analysis, scRNA-seq datasets from 4 RB samples in our prior cohort (GSE249995) [17] were integrated with publicly available scRNA-seq datasets from 5 normal retina samples (E-MTAB-7316) and 7 RB samples (GSE168434) [18, 19] using the Seurat R package (v4.0.3) [20]. Subsequently, unsupervised clustering analysis, dimensionality reduction, and cell type annotation were conducted according to established protocols [17], with cell type assignment based on known gene markers from previous studies representing various cell types [21–23]. Differentially expressed genes (DEGs) were defined with a significance threshold of *p* < 0.05. To characterize single-cell metabolic signatures, a computational pipeline was implemented following established procedures [24, 25]: gene expression quantification was performed by applying a log2 (TPM + 1) transformation; cell types containing fewer than 50 cells were excluded to ensure statistical reliability; metabolic gene sets and pathways were retrieved from the Kyoto Encyclopedia of Genes and Genomes (KEGG) database [26]; imputed expression values were used for t-SNE-based clustering to visualize cells with similar metabolic profiles; and quantitative metrics and algorithms were developed to identify cell type-specific metabolic programs and quantify pathway activity, enabling comprehensive assessment of metabolic activities at the single-cell level. Additionally, the Monocle3 software (v1.2.9) was utilized to infer pseudo-time trajectories [27], with DEGs between different states determined using the fit_models function and a *Q*-value threshold of <0.001.

### Lentiviral vector construction and transduction for B4GALT3 manipulation

To construct stable cell lines for overexpression and knockdown of human *B4GALT3*, the full-length CDS of B4GALT3 (NM_001199873) was cloned into the pCDH-CMV-MCS-COpGFP-T2A-Puro-3flag vector (PC-0731-001, synthesized by Guangzhou JIDAN Biotechnology Co., Ltd.) via EcoRI/XhoI digestion for overexpression. Two specific shRNA sequences targeting B4GALT3 (shB4GALT3-1: CCACATCTGTAGGACACTATA; shB4GALT3-2: GTGGTGAGGATGACGACATTG) were inserted into the pLKO.1 lentiviral vector (Addgene #8453) using AgeI/EcoRI sites for knockdown. HEK293T cells were co-transfected with the recombinant plasmids and packaging plasmids pSPAX2/pMD2G at a molar ratio of 1:1:1. After 8 h in serum-free medium, the transfection mixture was replaced with DMEM supplemented with 10% FBS. Viral supernatants were collected at 48 and 72 h post-transfection, filtered through 0.45-μm membranes. For lentiviral transduction, RB cells were pre-cultured in RPMI-1640 medium supplemented with 10% FBS and 1% penicillin-streptomycin, adjusted to a density of 1×10⁵ cells/mL, and infected with the respective viral particles at an MOI of 10 in the presence of 5 μg/mL polybrene. Negative control groups included cells transduced with empty vectors (pCDH-CMV-COpGFP-T2A-Puro for overexpression control and pLKO.1-puro for knockdown control). After 12 h, the medium was replaced with fresh RPMI-1640, and 72 h post-infection, puromycin-resistant colonies were selected using 1 μg/mL puromycin for 7 days with medium changes every 2 days.

### Total RNA extraction and RT-qPCR

Total RNA was isolated using the EZ-press RNA Purification Kit (EZBioscience, B0004D). cDNA was synthesized from 1 μg RNA with the Color Reverse Transcription Kit (EZBioscience, A001GQ). qPCR was performed in 10-μL reactions containing SYBR Green Master Mix, 0.5 μM primers, and cDNA template. Relative mRNA levels were normalized to GAPDH and expressed as fold changes relative to the control group. Primer sequences are provided in Supplementary Table S1.

### Western blotting and lectin pull-down assay

Cells were washed with PBS and lysed in RIPA Lysis Buffer (WB3100; NCM Biotech, Suzhou, China) supplemented with ProtLytic Protease and Phosphatase Inhibitor Cocktail (P002; NCM Biotech, Suzhou, China). Protein concentration was determined using the Pierce BCA Protein Assay Kit (23225; Thermo Fisher Scientific). Equal amounts of protein were mixed with Omni-Easy™ Protein Sample Loading Buffer (Denaturing, Reducing, 5×; Epizyme Biotech, Shanghai, China), denatured at 75 °C for 10 min, resolved by SDS-PAGE, and transferred to PVDF membranes (Millipore, USA). Membranes were blocked with 5% bovine serum albumin (BSA; Solarbio, China) in tris-buffered saline with 0.1% Tween-20 (TBST) for 1 h at room temperature, followed by overnight incubation at 4 °C with primary antibodies (details in Table S2). After three 10-min washes in TBST, membranes were incubated with HRP-conjugated Affinipure Goat Anti-Mouse IgG(H + L) (1:10,000, SA00001-1; ProteinTech) or HRP-conjugated Affinipure Goat Anti-Rabbit IgG(H + L) (1:10,000, SA00001-2; ProteinTech) for 1 h at room temperature. Following three additional 10-min washes in TBST, protein bands were visualized using enhanced chemiluminescence (Tanon, Shanghai, China) and imaged with the Tanon 5200 MultiImage System. For lectin pull-down assays, 200 mg of cell lysates were incubated with biotinylated Ricinus communis Agglutinin I (RCA I, RCA120; Vector Laboratories, Burlingame, CA) overnight at 4 °C. Subsequently, 50 μL of Streptavidin Agarose (P2159; Beyotime, China) was added, and the mixture was rotated for an additional 6 h at 4 °C. The pulled-down proteins were then subjected to Western blot analysis.

### Immunofluorescence (IF)

Paraffin-embedded tissue sections were deparaffinized in xylene, rehydrated through graded ethanol, and rinsed in distilled water. Heat-induced epitope retrieval was performed in citrate buffer (pH 6.0) using microwave heating. After cooling and washing in PBS, endogenous peroxidase activity was blocked with 3% H₂O₂, followed by blocking with 3% BSA. Sections were then sequentially incubated with primary antibodies (details in Table S2) overnight at 4 °C, followed by corresponding HRP-conjugated secondary antibodies and Tyramide Signal Amplification (TSA) fluorophores, with thorough PBS washing and antibody elution steps between each round of primary/secondary/TSA labeling to enable multiplexing. Nuclei were counterstained with DAPI. Finally, sections were mounted and whole-slide imaging was performed using a confocal microscope.

### Histopathological analysis

Paraffin-embedded tissue sections underwent deparaffinization in xylene, rehydration through graded ethanol, and distilled water rinsing. For Ki67 immunohistochemistry, antigen retrieval was performed in citrate buffer (pH 6.0) using microwave heating. After endogenous peroxidase blockade (3% H₂O₂) and protein blocking (3% BSA), sections were incubated with anti-Ki67 primary antibody (1:500, Servicebio GB111499) at 4 °C overnight, followed by HRP-conjugated secondary antibody. Ki67-positive cells were visualized with DAB chromogen and counterstained with hematoxylin. Quantification was performed by calculating the percentage of Ki67-positive cells in ≥3 random high-power fields (400×) per sample using ImageJ. Periodic Acid-Schiff (PAS) staining and hematoxylin & eosin (HE) staining followed standard protocols, with PAS-positive area percentage quantified similarly in high-power fields using ImageJ.

### Cell viability assay

Cell viability was assessed using the Cell Counting Kit-8 (CCK-8; Vazyme, A311-01). Y79 and WERI-Rb1 cells were seeded in 96-well plates at 5,000 cells/well. For proliferation analysis of shB4GALT3 and B4GALT3-OE cells, CCK-8 reagent (10 μL/well) was added at 24, 48, and 72 h after seeding, followed by 4 h incubation. For natural compound treatments, cells were exposed to compounds for 24 h before CCK-8 addition. Absorbance was measured at 450 nm. Viability inhibition and proliferation rates were calculated using GraphPad Prism 8.0, with all experiments performed in triplicate.

### EdU proliferation assay

Cell proliferation was assessed using the BeyoClick™ EdU Kit with Alexa Fluor 594 (C0078, Beyotime). Transfected cells were seeded on poly-D-lysine (PDL)-coated coverslips (6-well plates, 1×10⁵ cells/well) for 24 h, pulsed with 10 μM EdU for 2 h at 37 °C, then fixed in 4% PFA. After permeabilization with 0.1% Triton X-100 and PBS washes, cells were incubated with click reaction cocktail (100 μL, 30 min) and counterstained with Hoechst 33342. Fluorescence images were captured under consistent exposure settings (SOPTOP XD-RFL microscope) and quantified using ImageJ by calculating EdU⁺ nuclei (Alexa Fluor 594) as a percentage of total nuclei (Hoechst) across three random 400× fields per sample.

### RNA sequencing and bioinformatic analysis

Total RNA extracted from shB4GALT3- or shNC-treated WERI-Rb1 cells was subjected to paired-end sequencing on an Illumina NovaSeq 6000 platform (Novogene, Beijing) with 2×150 bp reads. Library quality was assessed using the Agilent 5400 system (Agilent, USA) and quantified via qPCR (1.5 nM). Raw FASTQ files were processed using Fastp (v0.23.4) to trim adapter sequences and filter low-quality bases [28]. High-quality reads were aligned to the GRCh38.p13 reference genome using STAR (v2.7.11b) and counted based on GENCODE v39 GTF annotations [29, 30]. Gene expression levels were estimated from alignment files using RSEM (v1.3.3), generating outputs for expected counts, transcripts per million (TPM), and fragments per kilobase per million mapped fragments (FPKM) [31]. Genes with zero TPM values were excluded from downstream analyses. Differential expression analysis was performed using the DESeq2 R package (v1.39.8) [32], and identified DEGs were sorted by *P*-values. Functional enrichment analysis, including Gene Ontology (GO) biological process (BP) and Kyoto Encyclopedia of Genes and Genomes (KEGG) pathway analyses, was conducted using DAVID with significance set at p < 0.05 (Huang et al., 2009). Additionally, Gene Set Enrichment Analysis (GSEA) was performed to further explore the functional implications of DEGs [33].

### Cell adhesion assay

24-well plates were coated with fibronectin (FN; Solarbio, F8180) diluted in sterile saline (5 μg/cm²), air-dried for 45 min at room temperature, and residual solution removed. For genetic manipulation studies, shB4GALT3- or B4GALT3-OE-transfected RB cells were seeded and allowed to adhere for 48 h. For pharmacological experiments, wild-type RB cells were pre-adhered for 24 h prior to compound treatment, followed by an additional 24 h incubation. All samples were fixed, stained with 0.1% crystal violet, and washed. Adherent cells were quantified in ≥3 random 200× fields per well.

### Co‑culture of ARPE‑19 and RB cells

To investigate the impact of retinoblastoma (RB) cells on the integrity of the outer blood–retinal barrier, we established a Transwell co‑culture model in 24‑well plates (Corning) using ARPE‑19 and WERI‑Rb1 cells. ARPE‑19 cells were seeded in the lower chamber in DMEM/F‑12 complete medium and allowed to adhere for 24 h, after which 0.8 µm‑pore Transwell inserts were introduced and WERI‑Rb1 cells—wild‑type, B4GALT3‑knockdown or B4GALT3‑overexpressing—were added to the upper chamber in RPMI‑1640 complete medium. Following 48 h of co‑culture, ARPE‑19 monolayers were subjected to immunofluorescence staining for ZO‑1 (Proteintech; Cat. No. 21773‑1‑AP) and occludin (Cell Signaling Technology; Cat. No. E6B4R) and parallel lysates were prepared for western blot analysis. To assess the roles of FAK and MMP2 in barrier disruption, inhibitors were added to the upper chamber 24 h after the start of co‑culture: Ilomastat (GM6001; S7157; Selleck Chemicals, USA) at 0.5 nM to block MMP activity, or PF‑573228 (S2013; Selleck Chemicals, USA) at 4 nM to inhibit FAK. Cells were incubated for an additional 24 h before fixation and protein extraction for immunofluorescence and western blotting as described above.

### Gelatin zymography

MMP activity was analyzed using a commercial kit (RTD6143, Real-Times Biotechnology). Conditioned media from wild-type, shB4GALT3, and B4GALT3-OE groups underwent non-reducing electrophoresis in gelatin-embedded gels (150 V). Gels were renatured in Renaturation Buffer, then incubated in Development Buffer at 37 °C for 4 h. After distilled water rinses, gels were stained with FastBlue solution (RT, 30 min-2h). Proteolytic activity manifested as cleared zones against blue-stained gelatin backgrounds.

### Structure-based virtual screening for B4GALT3-targeting compounds

A structure-based virtual screening approach was conducted to identify natural compounds with potential affinity for B4GALT3. The human B4GALT3 structure (AF-O60512-F1) predicted by AlphaFold was retrieved and prepared using the Protein Preparation Wizard in the Schrödinger Suite (v11.4). The TargetMol Bioactive Compound Library (T001; >18,000 natural products) was energy-minimized using LigPrep prior to screening. A hierarchical molecular docking workflow was employed, comprising high-throughput virtual screening (HTVS) of all compounds, followed by standard precision (SP) docking of the top 10% HTVS hits and extra precision (XP) docking of the top 10% SP hits. Ligands were ranked according to Glide XP scores, with more negative scores indicating stronger predicted binding affinity. The top 15 ligands were selected for subsequent experimental validation. Predicted protein–ligand interactions were analyzed and visualized using two- and three-dimensional models generated in PyMOL.

### Cellular thermal shift assay (CETSA)

For the cellular thermal shift assay (CETSA) in cell lysates [34], cells were harvested, washed once with ice-cold PBS, and resuspended in PBS supplemented with ProtLytic Protease and Phosphatase Inhibitor Cocktail (P002; NCM Biotech, Suzhou, China). Cells were lysed by three freeze–thaw cycles in liquid nitrogen, and the lysates were clarified by centrifugation at 20,000 × *g* for 20 min at 4 °C. The resulting supernatants were diluted in PBS and divided into two groups: one incubated with myricoside (100 µM) and the other with vehicle control for 30 min at room temperature. Samples (50 µL per aliquot) were then heated at the indicated temperatures (37, 41, 45, 49, 53, 57, 61, and 65 °C) for 5 min and subsequently cooled to room temperature for 3 min. After a final centrifugation at 20,000 × *g* for 20 min at 4 °C, supernatants containing soluble proteins were collected for immunoblot analysis.

### PI/Hoechst dual staining for apoptosis assessment

To evaluate the effect of myricoside on RB cell apoptosis, cells were seeded onto PDL‑coated six‑well plates and treated with varying concentrations of myricoside for 24 h. After treatment, nuclei were stained with Hoechst 33342 to visualize total cells, while propidium iodide (PI) was used to label non‑viable cells. Images were acquired under a fluorescence microscope at 400× magnification, capturing three randomly selected fields per well. The proportion of PI‑positive cells was quantified using ImageJ software.

### Terminal deoxynucleotidyl transferase‑dUTP nick end labeling (TUNEL) assay

Paraffin‑embedded tissue sections were deparaffinised and processed for apoptosis detection using the One‑step TUNEL In Situ Apoptosis Kit (Green, Elab Fluor® 488; Elabscience) according to the manufacturer’s instructions. TUNEL‑positive apoptotic cells were quantified by analyzing three randomly selected fields containing viable tumor cells within each xenograft section. The percentage of apoptotic cells per tumor‑bearing eye was calculated as the mean proportion of TUNEL‑positive nuclei relative to the total number of nuclei in each field.

### Animal studies

For orthotopic xenograft experiments, female BALB/c nude mice (4–6 weeks old; 18–20 g body weight; Zhuhai BesTest Bio-Tech Co., Zhuhai, China) were housed under specific pathogen‑free (SPF) conditions with controlled temperature and humidity. RB xenografts were established as previously described [35, 36]. Briefly, WERI‑Rb1 cells (wild‑type, B4GALT3 knockdown or B4GALT3 overexpression; 1 × 10⁵ cells in 1 µL PBS) were injected into the vitreous cavity of the right eye using a 33 G Hamilton syringe, with the contralateral eye left untreated as an internal control (*n* = 8 mice per group). Tumor formation was assessed 30 days post‑injection. To evaluate the therapeutic effect of myricoside, intravitreal injections of myricoside (100 nM) or vehicle (DMSO) were administered 14 days after xenograft implantation (*n* = 8 mice per group); eyes were collected 28 days after xenograft implantation. Following enucleation, five eyes per group were fixed and paraffin‑embedded for histological analysis, and the remaining three were processed for protein extraction.

### Statistics analysis

All data are presented as the mean ± standard deviation (SD) from at least three independent experiments or biological replicates. Individual data points represent biological replicates. Statistical analyses were performed using GraphPad Prism (v8.0.2; GraphPad, Inc., La Jolla, CA, USA). Comparisons between two groups were conducted using an unpaired, two‑tailed Student’s *t*‑test, and one‑way ANOVA was applied for multiple group comparisons. RNA‑seq data were analyzed using the DESeq2 package in R [32]. *P*-values < 0.05 were considered statistically significant. Significance levels are indicated in the figure legends.

## Results

### Increased expression of B4GALT3 in RB

**Fig. 1:**
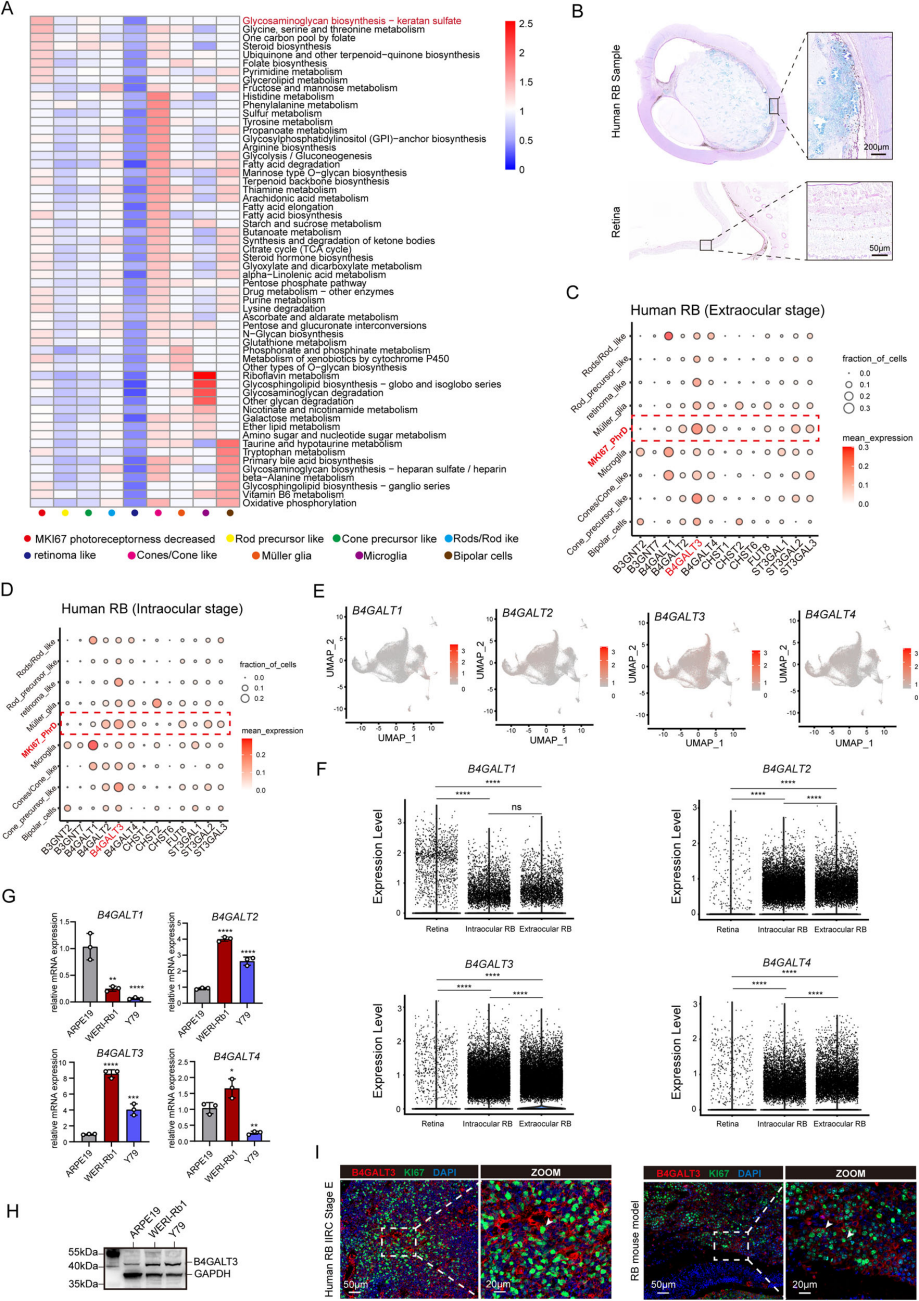
Increased expression of B4GALT3 in retinoblastoma. **A** Metabolic pathway activities across cell types in integrated retinoblastoma (RB) datasets (4 RB samples from GSE249995 and 7 from GSE168434). Pathways with non-significant activity (permutation test, *P* > 0.05) are shown as blank. **B** AB-PAS staining of a human RB paraffin section (International Intraocular Retinoblastoma Classification [IIRC] stage E). **C**, **D**. Dot plots showing normalized expression of genes involved in the glycosaminoglycan biosynthesis–keratan sulfate pathway in different cell types from extraocular (**C**) and intraocular (**D**) RB samples. **E** UMAP visualization of *B4GALT1*−*4* expression in RB cells. **F** Comparison of *B4GALT* family gene expression across retina, intraocular RB, and extraocular RB based on scRNA-seq data. **G**, **H** qPCR (**G**) and western blot (**H**) analyses demonstrating significantly elevated B4GALT3 mRNA and protein levels in RB cell lines (WERI-Rb1 and Y79) compared to human retinal pigment epithelial cells (ARPE-19). **I** Immunofluorescence co-staining of B4GALT3 and Ki67 in proliferative tumor regions of IIRC stage E RB and orthotopic xenografts. Data are presented as mean ± SD from 3 independent experiments. Statistical significance in (**G**) was determined by one-way ANOVA. ^*^P < 0.05; ^**^P < 0.01; ^***^P < 0.001; ^****^P < 0.0001.

Single‑cell RNA‑seq datasets from RB samples were integrated to assess metabolic pathway activity across distinct cellular subpopulations, including MKI67⁺ photoreceptor-decreased (MKI67⁺ PhrD) cells, cone precursor‑like cells, rod precursor‑like cells, cones/cone‑like cells, rods/rod‑like cells, müller glia, microglia, bipolar cells, and retinoma‑like cells. The MKI67⁺ PhrD population refers to the proliferative RB cell cluster. These cells exhibit high expression of proliferation-associated genes such as *MKI67*, *TOP2A*, *UBE2C*, *BIRC5*, and *TPX2*, accompanied by markedly decreased expression of photoreceptor-related genes, reflecting the characteristic loss of photoreceptor differentiation during RB progression [15, 19]. Pathway enrichment analysis revealed that MKI67^+^ PhrD cells exhibited significant activation of the glycosaminoglycan biosynthesis–keratan sulfate pathway (Fig. 1A). This pathway was largely inactive in non-malignant subtypes, implicating its potential association with tumor aggressiveness. Supporting this, AB–PAS staining revealed prominent glycosaminoglycan accumulation in intraocular RB tissues—particularly in regions adjacent to the scleral wall—whereas no evident glycosaminoglycan deposition was detected in normal retina (Fig. 1B).

Focusing on genes within this pathway, *B4GALT3* emerged as the top candidate with the highest expression in MKI67^+^ PhrD cells at both intraocular and extraocular stages (Fig. 1C, D). Additional analysis showed that *B4GALT3*-positive cells were the most abundant among the *B4GALT1–4* family in tumor cells (Fig. 1E), with expression levels markedly elevated in both intraocular and extraocular RB tissues compared to normal retina (Fig. 1F). In the pseudotemporal progression of MKI67^+^ PhrD cells, *B4GALT1* and *B4GALT2* exhibit low and stable expression, while *B4GALT3* and *B4GALT4* show upregulated expression and increased dispersibility (Fig. S1). WERI-Rb1 and Y79 cells showed higher B4GALT3 mRNA and protein levels than ARPE-19 (Fig. 1G, H). Furthermore, immunofluorescence in IIRC stage E RB and orthotopic xenografts demonstrated co-localization of B4GALT3 with Ki67 in proliferating tumor cells, further implicating B4GALT3 in the malignant compartment (Fig. 1I).

### B4GALT3 knockdown disrupts β1-integrin glycosylation and cell adhesion in RB

**Fig. 2:**
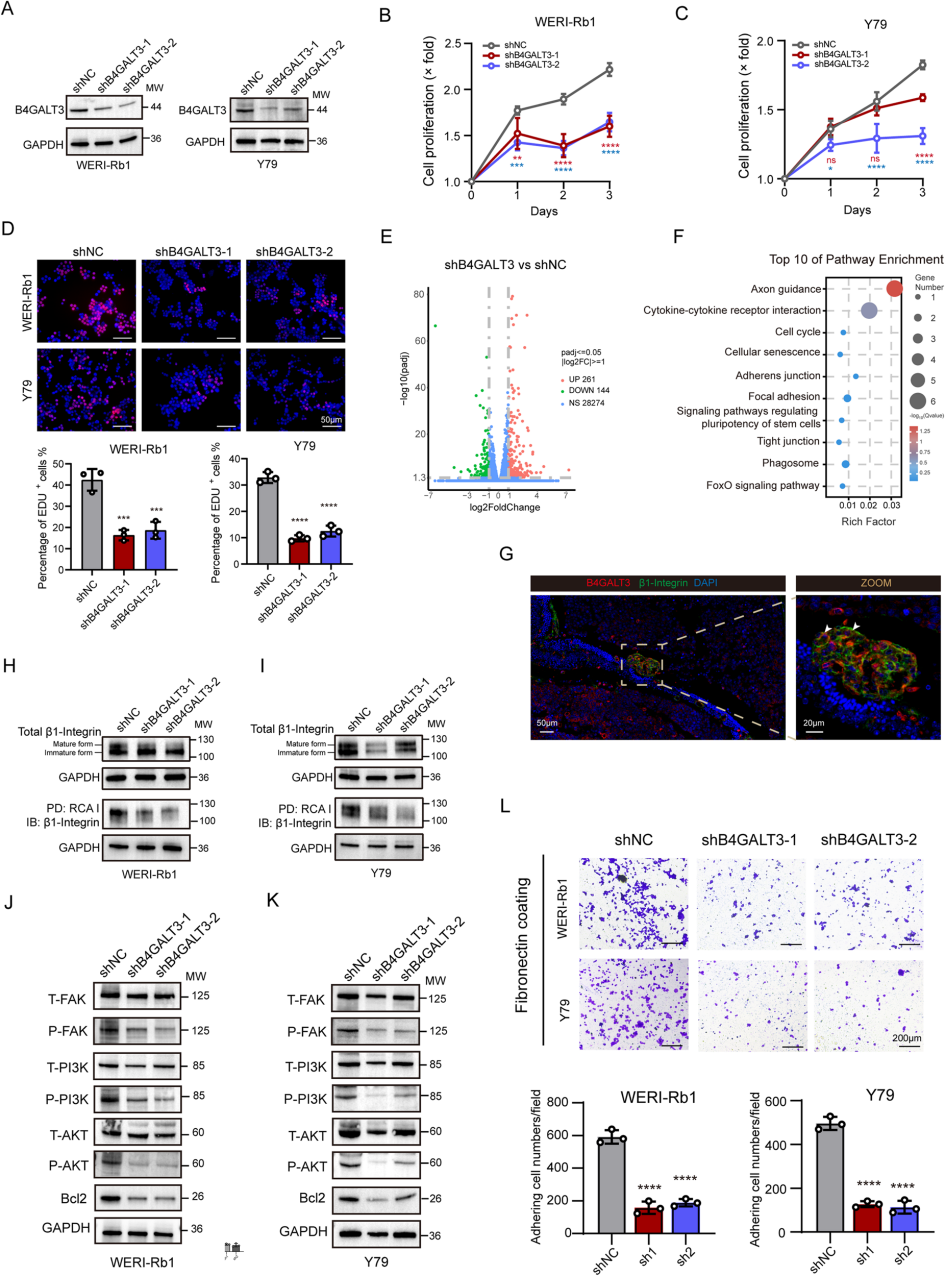
B4GALT3 knockdown disrupts β1-integrin glycosylation and cell adhesion in retinoblastoma. **A** Western blot analysis showing decreased B4GALT3 protein levels in WERI-Rb1 and Y79 cells upon knockdown using two independent shRNAs. **B**, **C** CCK-8 proliferation assays in RB cell lines WERI-Rb1 (**B**) and Y79 (**C**) following B4GALT3 knockdown with shRNA for 24–72 h. **D** Representative images and quantification of EdU incorporation assay in RB cell lines after B4GALT3 knockdown for 48 h. **E** Volcano plot depicting differentially expressed genes (DEGs) from RNA-seq analysis comparing WERI-Rb1 cells treated with control shRNA (shNC) and shB4GALT3. **F** Top 10 enriched pathways based on KEGG analysis of downregulated DEGs in WERI-Rb1 cells following B4GALT3 knockdown. **G** Representative immunofluorescence (IF) staining images demonstrating co-localization of β1-integrin and B4GALT3 in orthotopic xenograft sections. **H**, **I** Analysis of B4GALT3-modified glycosylation of β1-integrin in RB cells. Cell lysates from WERI-Rb1 (H) and Y79 (I) cells were subjected to RCA I pull-down (PD) followed by Western blot with an anti-β1-integrin antibody. **J**, **K** Western blot analysis showing alterations in the FAK-PI3K-AKT signaling pathway in WERI-Rb1 (**J**) and Y79 (**K**) cells following B4GALT3 knockdown. **L** Representative images and quantification of fibronectin adhesion assay in RB cells following B4GALT3 knockdown. Data are presented as mean ± SD from three independent experiments. Statistical significance in (**B**, **C**, **D**, **L**) was determined by one-way ANOVA. *ns*, no statistical difference; ^*^P < 0.05; ^**^P < 0.01; ^***^P < 0.001; ^****^P < 0.0001.

Given the marked upregulation of B4GALT3 in RB, its functional relevance was investigated through stable downregulation in RB cell lines. Western blot analysis confirmed effective downregulation of B4GALT3 in both WERI‑Rb1 and Y79 cells using two independent shRNAs (Fig. 2A). CCK-8 and EdU incorporation assays revealed that B4GALT3 downregulation significantly suppressed cell proliferation (Fig. 2B–D). Transcriptomic profiling (RNA-seq) comparing shB4GALT3 and shNC WERI‑Rb1 cells identified 261 upregulated and 144 downregulated genes (Fig. 2E). KEGG pathway analysis of downregulated genes in shB4GALT3-treated cells revealed significant enrichment in various cell adhesion-related pathways, including adherens junction, focal adhesion, and tight junction (Fig. 2F). Consistently, heatmaps showed broad downregulation of adhesion-associated genes such as *CDH2*, *CDH18*, *CLDN1*, *CLDN19*, *LAMA5*, and *LAMB1*, as well as glycosaminoglycan biosynthesis–related genes including *CHST* family members, *EXT2*, *CSPG4*, and *GPC1* in B4GALT3-knockdown cells (Fig. S2). Conversely, upregulated genes were enriched in cytokine–cytokine receptor interaction, lysine degradation, and Wnt signaling (Fig. S3).

Considering β1-integrins are critical cell–ECM receptors involved in cell survival, migration, and invasion, and their glycosylation is known to modulate these functions [37–39], we next examined their association with B4GALT3. Immunofluorescence staining of orthotopic xenografts revealed co‑expression of B4GALT3 and β1-integrin within tumor cells (Fig. 2G). RCA-I lectin pull-down assays demonstrated that B4GALT3 knockdown markedly reduced RCA-I-bound β1-integrin in both cell lines (Fig. 2H, I). Notably, this effect was restricted to the 130-kDa mature isoform, indicating selective modification of the functionally active β1-integrin. Concordantly, B4GALT3 knockdown impaired downstream β1-integrin–FAK signaling, as evidenced by decreased FAK phosphorylation and consequent suppression of PI3K/AKT activation and BCL2 expression (Fig. 2J, K). Functionally, fibronectin adhesion assays confirmed that B4GALT3 knockdown impaired cell adhesion to fibronectin (Fig. 2L), further supporting the critical role of B4GALT3-mediated galactosylation in integrin–ECM engagement in RB cells.

### B4GALT3 overexpression promotes RB cell proliferation and adhesion

**Fig. 3:**
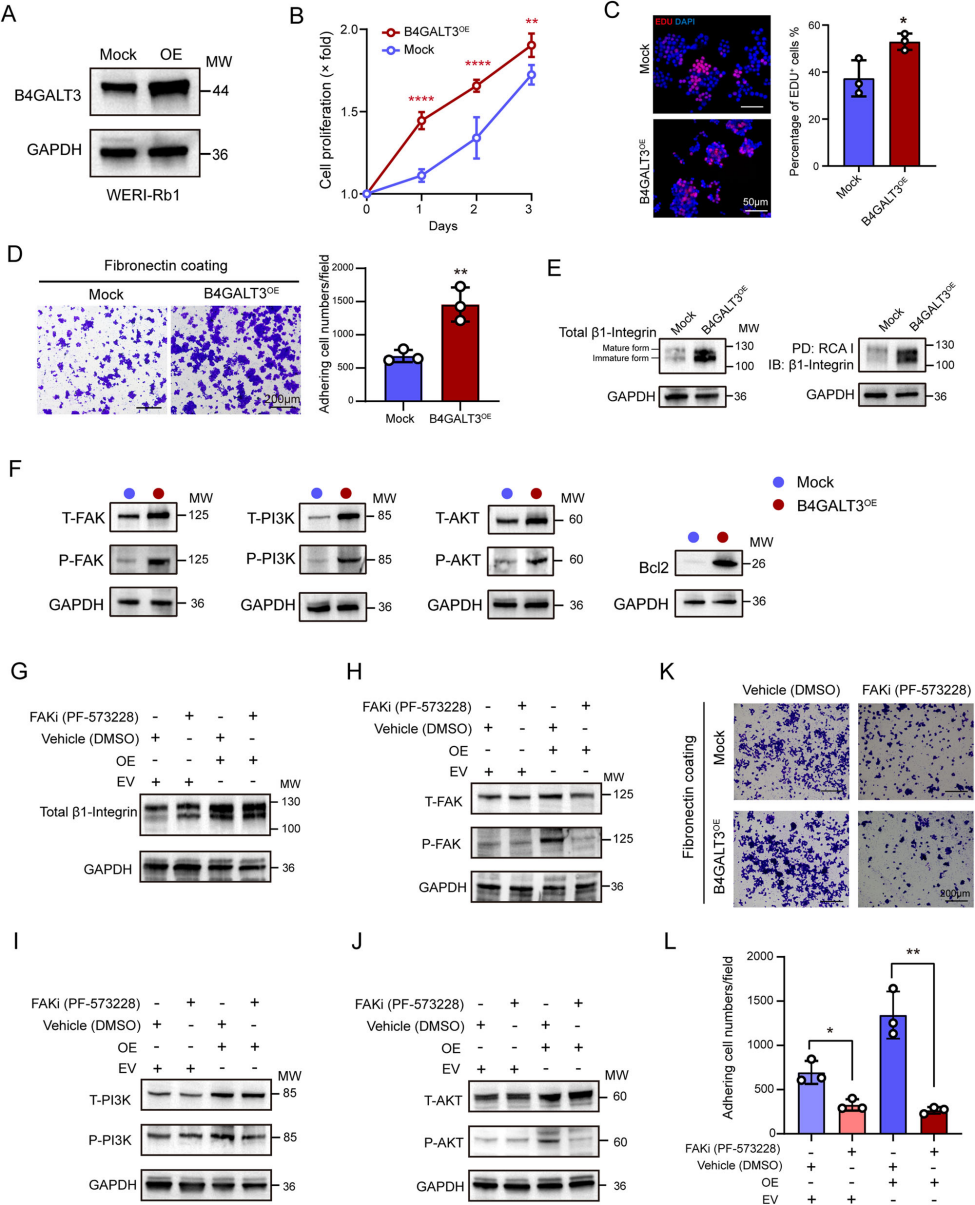
B4GALT3 overexpression promotes RB cell proliferation and adhesion. **A** Western blot analysis showing increased B4GALT3 protein levels in WERI-Rb1 cells following B4GALT3 overexpression. **B** CCK-8 proliferation assays in WERI-Rb1 cells following B4GALT3 overexpression for 24–72 h. **C** Representative images and quantification of EdU incorporation assay assessing proliferation in WERI-Rb1 cells after B4GALT3 overexpression for 48 h. **D** Representative images and quantification of fibronectin adhesion assay in WERI-Rb1 cells following B4GALT3 overexpression. **E** Analysis of B4GALT3-overexpression-induced glycosylation of β1-integrin in WERI-Rb1 cells. **F** Western blot analysis showing alterations in the FAK-PI3K-AKT signaling pathway in WERI-Rb1 cells following B4GALT3 overexpression. **G** Western blot analysis showing alterations of β1-integrin in B4GALT3-overexpressing WERI-Rb1 cells treated with FAK inhibition. **H**–**J** Western blot analysis showing alterations of the FAK-PI3K-AKT signaling pathway in B4GALT3-overexpressing WERI-Rb1 cells treated with FAK inhibition. Quantification of total FAK (tFAK) and phosphorylated FAK (pFAK) (**H**), total PI3K (tPI3K) and phosphorylated PI3K (pPI3K) (**I**), and total AKT (tAKT) and phosphorylated AKT (pAKT) (**J**). **K**, **L** Representative images (**K**) and quantification (**L**) of fibronectin adhesion assay in B4GALT3-overexpressing WERI-Rb1 cells treated with FAK inhibitor. Data are presented as mean ± SD from three independent experiments. Statistical significance in (**B**, **C**, **D**, **L**) was determined by a two-tailed unpaired *t*-test. *ns*, no statistical difference; ^*^P < 0.05; ^**^P < 0.01; ^****^P < 0.0001.

Building on the loss-of-function findings, we next examined the effect of B4GALT3 overexpression. Stable transfection of WERI‑Rb1 cells with a B4GALT3 expression vector resulted in elevated protein levels (Fig. 3A). Compared to controls, B4GALT3 overexpression increased cell proliferation and the proportion of EdU-positive cells (Fig. 3B, C). Additionally, fibronectin adhesion assays revealed that B4GALT3 overexpression promoted cell adhesion to fibronectin (Fig. 3D). In contrast to the knockdown group, B4GALT3 overexpression not only increased the total protein levels of β1-integrin but also enhanced its glycosylation state, as shown by RCA-I lectin binding (Fig. 3E). Moreover, overexpression of B4GALT3 activated the FAK-PI3K-AKT signaling pathway, as evidenced by the increased levels of both total and phosphorylated FAK, PI3K, and AKT (Fig. 3F).

To further dissect the role of FAK in B4GALT3-mediated signaling and adhesion, we treated B4GALT3-overexpressing cells with the FAK inhibitor PF-573228. FAK inhibition did not alter total β1-integrin levels but effectively blocked B4GALT3-induced FAK, PI3K, and AKT phosphorylation (Fig. 3G–J). Functionally, fibronectin adhesion assays demonstrated that FAK inhibition significantly reversed the B4GALT3-mediated enhancement of cell adhesion (Fig. 3K, L). Collectively, these results indicate that B4GALT3 promotes RB cell adhesion by enhancing β1-integrin glycosylation and activating the FAK–PI3K–AKT signaling axis.

### B4GALT3 promotes RB invasion by modulating the FAK-MMP2 axis

**Fig. 4:**
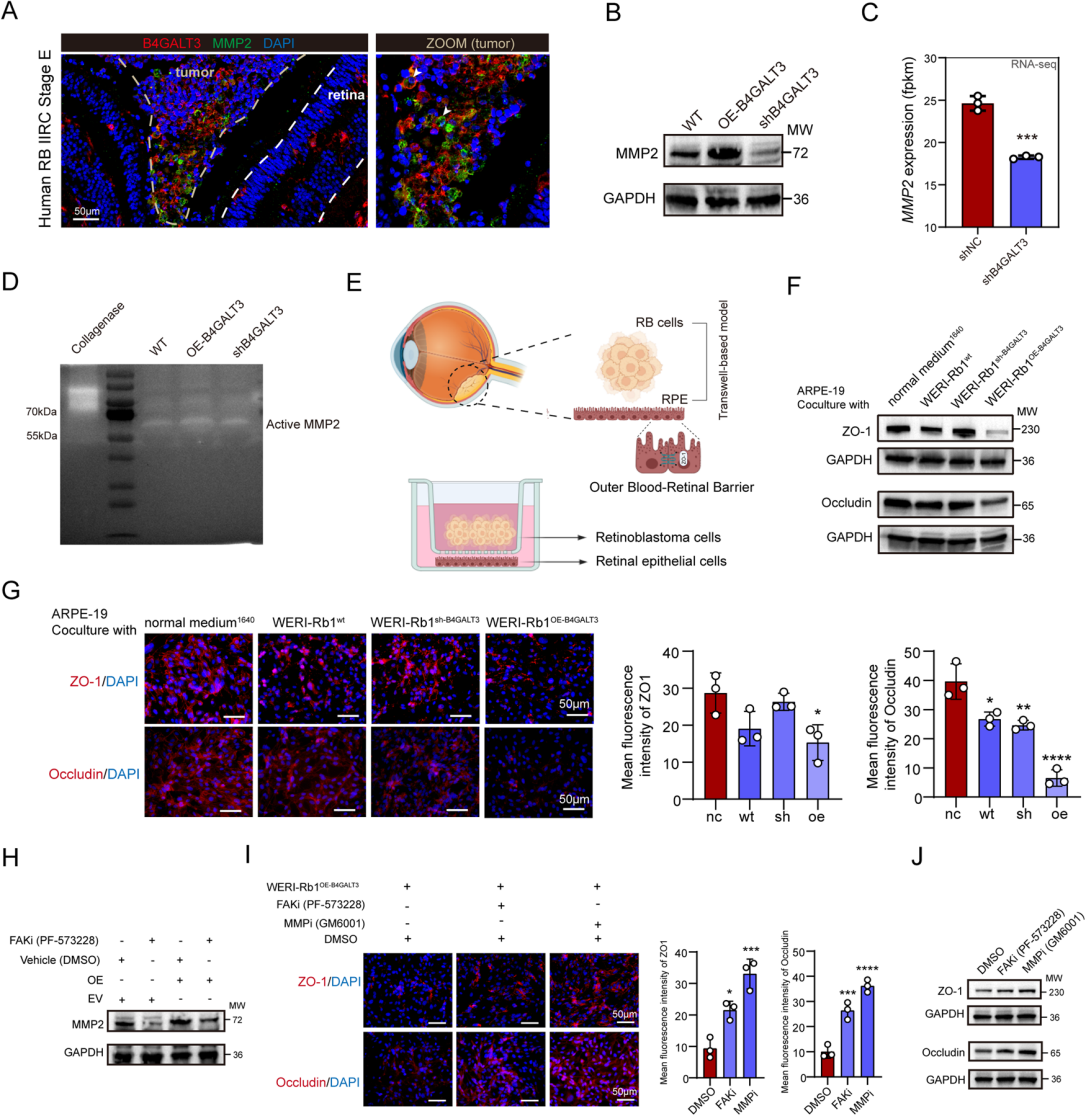
B4GALT3 Promotes RB Invasion by Modulating the FAK-MMP2 Axis. **A** Representative immunofluorescence (IF) staining image demonstrating co-localization of MMP2 and B4GALT3 in human IIRC stage E RB sections. **B** Western blot analysis of MMP2 expression in WERI-Rb1 cells following B4GALT3 knockdown or overexpression. **C** MMP2 mRNA expression in RNA-seq data of WERI-Rb1 cells treated with control shRNA (shNC) or shB4GALT3. **D** Gelatin zymography assay showing the levels of active MMP2 in the supernatants of WERI-Rb1 cells with B4GALT3 knockdown or overexpression. **E** Schematic diagram of a co-culture system of RB cells and ARPE-19 retinal epithelial cells to model tumor invasion across the outer blood–retinal barrier. **F** Western blot analysis of ZO-1 and occludin in ARPE-19 cells co-cultured with WERI-Rb1 cells under B4GALT3 modulation (knockdown or overexpression). **G** Representative immunofluorescence images and quantification of ZO-1 and occludin in ARPE-19 co-cultures with B4GALT3-modulated WERI-Rb1 cells. **H** Western blot analysis of MMP2 expression in B4GALT3-overexpressing WERI-Rb1 cells treated with a FAK inhibitor. **I** Representative images and quantification of ZO-1 and occludin immunofluorescence staining in ARPE-19 cells co-cultured with B4GALT3-overexpressing WERI-Rb1 cells, following FAK and MMP inhibition. **J** Western blot analysis of ZO-1 and occludin in ARPE-19 co-cultures under the same conditions as in (**I**). Data are presented as mean ± SD from three independent experiments. Statistical significance was determined by a two-tailed unpaired *t*-test for (**C**), and one-way ANOVA for (**G**) and (**I**). *ns*, no statistical difference; ^*^P < 0.05; ^**^P < 0.01; ^***^P < 0.001; ^****^P < 0.0001.

Matrix metalloproteinases, particularly MMP-2 and MMP-9, are well-known mediators of extracellular matrix degradation and are strongly associated with optic nerve invasion and advanced clinicopathologic stages in RB. [40] Prior studies have shown that MMP-2 is frequently upregulated in invasive RB tissues and functionally contributes to tumor cell infiltration, making it a reliable molecular indicator of RB invasive potential. [41] To investigate the role of B4GALT3 in RB invasion, we first examined clinical RB specimens and observed a prominent co-localization of B4GALT3 and MMP2 in tumor cells adjacent to the retina (Fig. 4A), suggesting a spatial correlation that may support a functional relationship. In the WERI-Rb1 cells, MMP2 protein levels were significantly elevated in B4GALT3- OE cells and reduced in B4GALT3-sh cells (Fig. 4B). RNA-seq data further confirmed downregulation of MMP2 mRNA upon B4GALT3 knockdown (Fig. 4C). Gelatin zymography revealed that active MMP2 levels were elevated in B4GALT3-OE cells, supporting the notion that B4GALT3 modulates MMP2 activity (Fig. 4D).

To model RB invasion across the outer blood-retinal barrier, we established a co-culture system of RB cells and ARPE-19 retinal epithelial cells (Fig. 4E). Notably, co-culture with B4GALT3-OE RB cells led to a pronounced downregulation of the tight junction proteins ZO-1 and Occludin in ARPE-19 cells (Fig. 4F, G). Given B4GALT3’s regulation of FAK signaling, we further examined the potential involvement of FAK in MMP2 regulation. Western blotting (Fig. 4H) revealed that FAK inhibition reduced MMP2 expression in B4GALT3-OE cells. Moreover, either FAK or MMP2 inhibition effectively restored ZO-1 and Occludin expression and barrier continuity in the co-culture model (Fig.4 I, J), implicating a FAK–MMP2 pathway in B4GALT3-driven RB invasion.

### B4GALT3 promotes retinoblastoma invasion in vivo

**Fig. 5:**
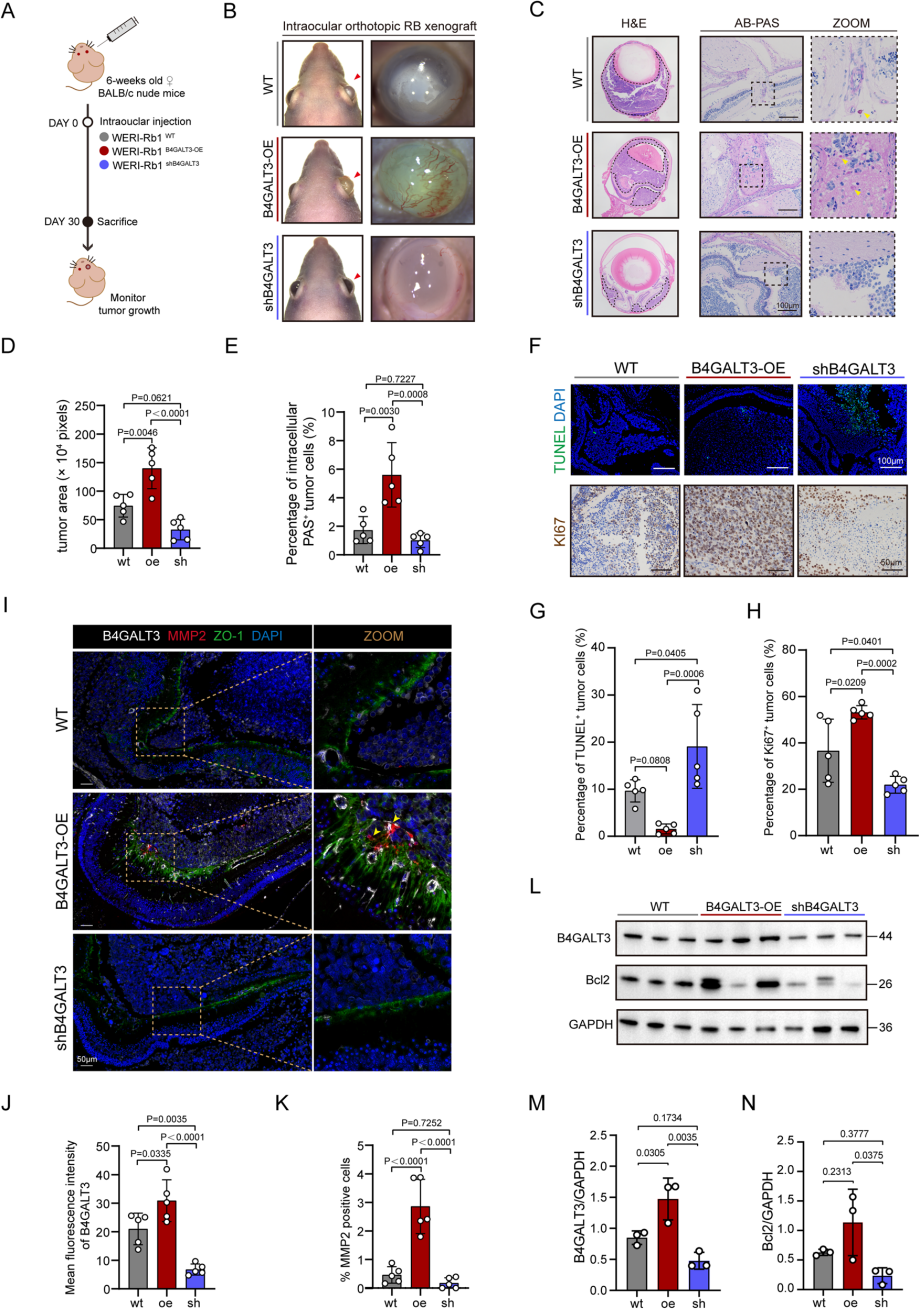
B4GALT3 promotes retinoblastoma growth and invasion in vivo. **A** Schematic illustration of the orthotopic intraocular xenograft model. **B** Representative eye views at 30 days post-xenograft from each experimental group. **C** H&E and PAS staining of enucleated eyes from control, B4GALT3 knockdown (shB4GALT3), and overexpression (B4GALT3-OE) groups. **D** Quantification of intraocular tumor area in each group. **E** Percentage of PAS-positive cells in each group. **F** Representative images of TUNEL and Ki67 staining in enucleated eyes. **G** Quantification of TUNEL-positive cells. **H** Quantification of Ki67-positive cells. **L** Immunofluorescence staining of MMP2, ZO-1, and B4GALT3 in xenograft tissues. **J** Quantification of B4GALT3 fluorescence intensity. **K** Quantification of MMP2-positive cell percentage. Western blot analysis of B4GALT3 and BCL2 expression in each group (**L**), with corresponding quantification (**M**, **N**). Data are presented as mean ± SD, based on five replicates for panels (**D**, **E**, **G**, **H**, **J**, **K**) and three replicates for panels (**M**, **N**). Statistical significance was determined using one-way ANOVA. Exact *P*-values are indicated in the corresponding figures.

To validate the oncogenic role of B4GALT3 in vivo, an orthotopic intraocular xenograft model was established (Fig. 5A). Gross examination revealed that eyes bearing B4GALT3-overexpressing tumors developed pronounced proptosis and extensive corneal neovascularization, whereas B4GALT3 knockdown markedly suppressed tumor progression (Fig. 5B). Histopathological assessment via H&E staining demonstrated that B4GALT3-overexpressing (OE) tumors exhibited enhanced invasive morphology and significantly increased intraocular tumor burden **(**Fig. 5C, D). AB-PAS staining indicated a higher proportion of PAS-positive cells in the B4GALT3-OE tumors, suggesting alterations in mucin or glycogen-rich secretory phenotypes associated with increased invasion (Fig. 5 C, E). Proliferation and apoptosis assays showed more Ki67^+^ cells and fewer TUNEL^+^ cells in B4GALT3-OE tumors compared to knockdown tumors (Fig. 5F–H). Immunofluorescence confirmed co-localization of B4GALT3 and MMP2 with discontinuous ZO-1 in the B4GALT3-OE group, indicating tight junction disruption (Fig. 5I–K). Moreover, colocalization of ITGB1 and p-FAK was increased in B4GALT3-overexpressing tumors but reduced in B4GALT3-knockdown tumors (Fig. S4A). Western blotting further confirmed elevated B4GALT3 and Bcl2 expression in B4GALT3-OE tumors and reduced levels in B4GALT3-sh tumors (Fig. 5L–N).

### B4GALT3 inhibitor myricoside suppresses RB progression

**Fig. 6:**
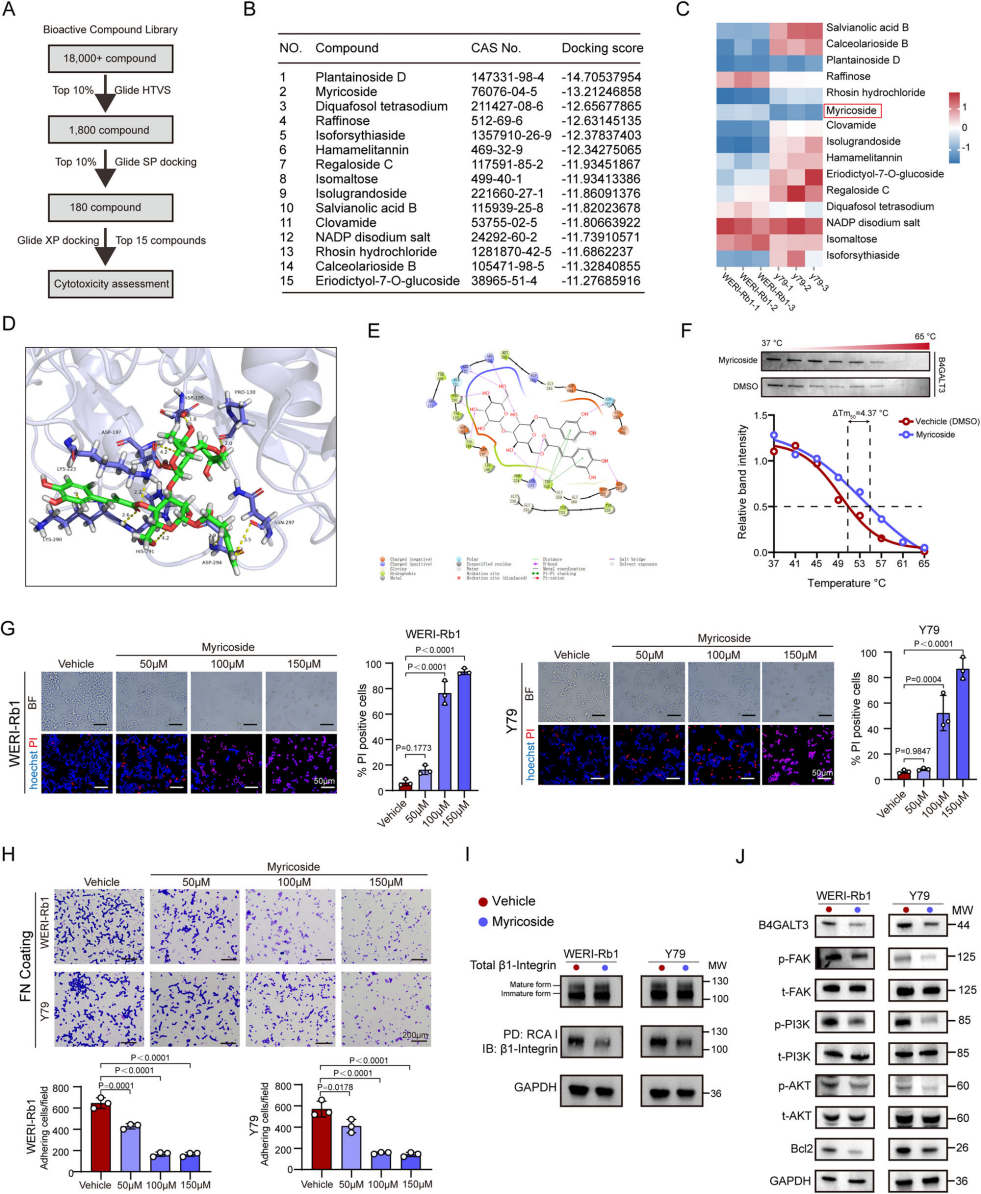
Identification and validation of a B4GALT3 inhibitor targeting RB cell viability and signaling. **A** Schematic workflow of high-throughput virtual screening (HTVS) for identifying potential B4GALT3 inhibitors. **B** Docking scores of the top 15 candidate compounds identified from the HTVS. **C** Quantification of cell viability in RB cell lines (WERI-Rb1 and Y79) treated with the top 15 candidate compounds (100 μM) for 24 h. **D**, **E** In silico docking of Myricoside into the active site of human B4GALT3 protein (**D**), highlighting the detailed molecular interactions within the binding pocket (**E**). **F** Cellular thermal shift assay (CETSA) curves showing thermal stabilization of B4GALT3 protein in RB cell lysates with or without myricoside treatment (100 μM). **G**, **H** Representative images and quantification of (**G**) propidium iodide (PI)-positive cells and (H) fibronectin-adherent cells in RB cell lines treated with increasing concentrations of myricoside for 48 h. **I** Western blot analysis showing alterations in β1-integrin glycosylation in RB cells treated with myricoside (100 μM). **J** Western blot analysis of the FAK–PI3K–AKT signaling pathway in RB cells following myricoside treatment (100 μM). Data are presented as mean ± SD from three independent experiments. Statistical significance was determined by one-way ANOVA for (**G**) and (**H**). Exact *P*-values are indicated in the corresponding figures.

Given the lack of clinically available B4GALT3 inhibitors, we performed structure-based virtual screening of an 18,000-compound library (Fig. 6A). Sequential docking identified 15 top candidates for validation (Fig. 6B). Among these, myricoside showed strong predicted binding affinity and significant cytotoxicity in RB cells (Fig. 6C). Molecular docking revealed that myricoside occupies the catalytic pocket of B4GALT3, forming hydrogen bonds with key residues (Asp195, Asp197, His291, Asp294) and hydrophobic interactions (Fig. 6D–E). To confirm this interaction, cellular thermal shift assays (CETSA) demonstrated that myricoside significantly enhanced the thermal stability of B4GALT3, indicative of direct compound-protein binding (Fig. 6F). Functionally, myricoside induced dose-dependent RB cell death (Fig. 6G), reduced fibronectin adhesion (Fig. 6H), decreased β1-integrin galactosylation (Fig. 6I), and suppressed the FAK–PI3K–AKT–Bcl2 axis (Fig. 6J), mimicking the effects of B4GALT3 knockdown.

**Fig. 7:**
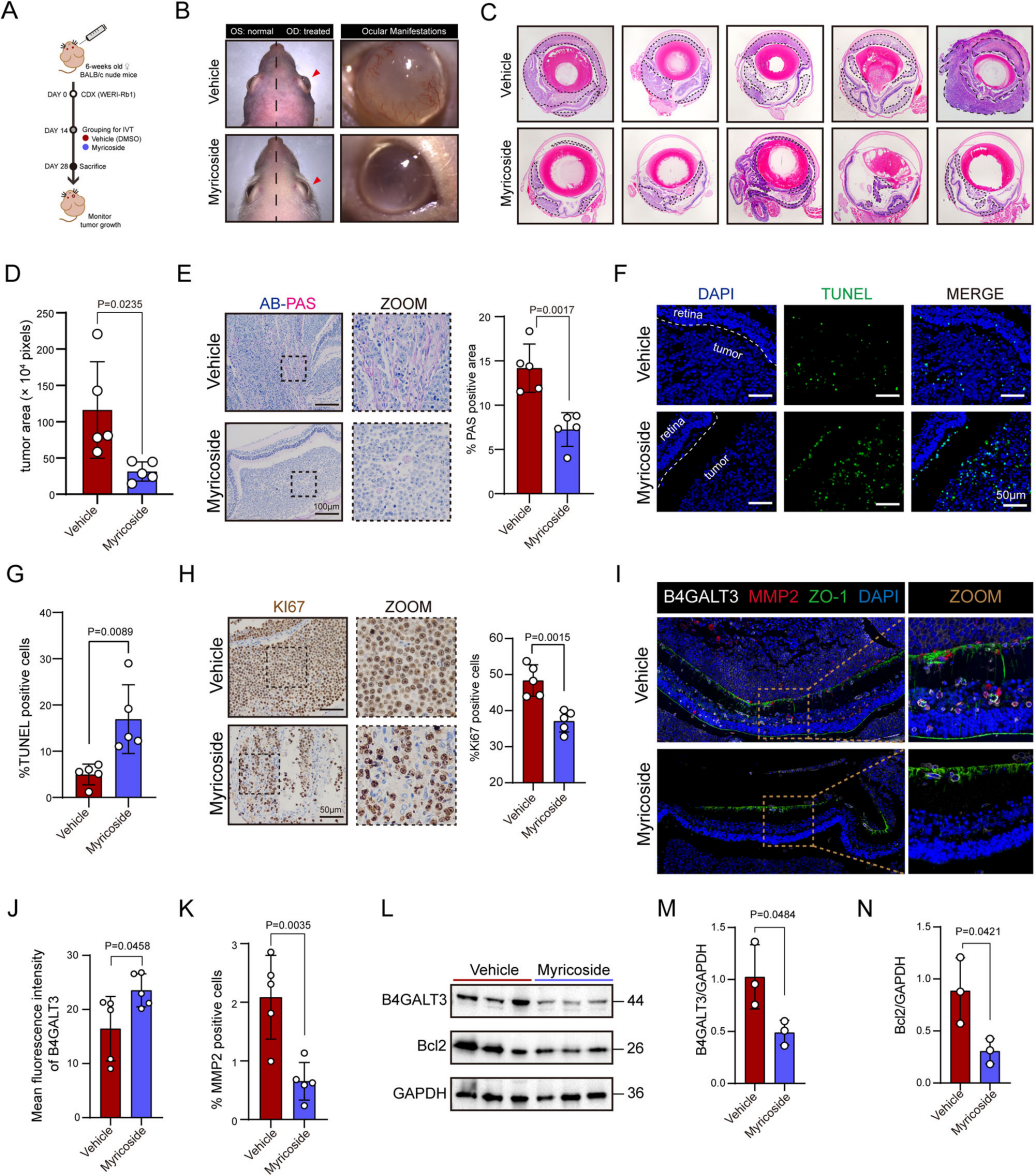
Myricoside treatment inhibits retinoblastoma growth and invasion in vivo. **A** Schematic illustration of the orthotopic intraocular xenograft model showing the myricoside treatment regimen. **B** Representative eye views at 28 days post-xenograft from vehicle and myricoside-treated groups. **C** H&E staining of enucleated eyes from vehicle and myricoside-treated groups. **D** Quantification of intraocular tumor area in enucleated eyes from each group. **E** AB-PAS staining and quantification of PAS-positive cells in xenografts from each group. **F**, **G** Representative TUNEL staining (**F**) and quantification of TUNEL-positive cell percentage (**G**) in enucleated eyes from each group. **H** Representative Ki67 staining and quantification of Ki67-positive cells in xenografts from each group. **I** Immunofluorescence staining of MMP2, ZO-1, and B4GALT3 in xenograft tissues. **J** Quantification of B4GALT3 fluorescence intensity. **K** Quantification of MMP2-positive cells in xenograft tissues. **L** Western blot analysis of B4GALT3 and BCL2 expression in each treatment group. Quantification of Western blot data for B4GALT3 (**M**) and BCL2 (**N**) expression. Data are presented as mean ± SD, based on five replicates for panels (**D**, **E**, **G**, **H**, **J**, **K**) and three replicates for panels (**M**, **N**). Statistical significance was determined using a two-tailed unpaired *t*-test. Exact *P*-values are indicated in the corresponding figures.

In an orthotopic xenograft model, intravitreal administration of myricoside attenuated proptosis and corneal neovascularization, and significantly reduced tumor size and invasiveness (Fig. 7A–D). Myricoside treatment significantly decreased the proportion of PAS-positive mucin-rich cells (Fig. 7E), increased apoptotic cell numbers as shown by TUNEL staining (Fig. 7F, G), and reduced the fraction of proliferating Ki67^+^ cells (Fig. 7H). Immunofluorescence co-staining showed reduced B4GALT3-MMP2 co-localization and restoration of ZO-1 integrity in myricoside-treated tumors (Fig. 7I). Consistently, myricoside treatment markedly decreased the number of ITGB1/p-FAK double-positive cells compared with the vehicle group (Fig. S4B). Quantitative fluorescence intensity analysis confirmed reduced B4GALT3 and MMP2 signals in treated tumors (Fig. 7J, K). Western blotting further confirmed that myricoside treatment downregulated B4GALT3 and Bcl2 levels in vivo (Fig. 7L–N). Importantly, intraocular Myricoside treatment did not affect retinal morphology or induce apoptosis, as reflected by comparable ganglion cell layer (GCL) cell numbers and TUNEL-positive cell counts between vehicle- and treated eyes (Fig. S5).

## Discussion

Given the poor prognosis in advanced RB, the development of effective eye‑preserving therapies is imperative. Our previous study has demonstrated that tumor metabolic heterogeneity plays a pivotal role in RB progression [25, 35, 42]. Here, we uncover B4GALT3 as a key driver of RB malignancy via dual regulation of β1‑integrin signaling. Integrating single‑cell RNA‑seq, patient specimens and functional assays, we show that B4GALT3 is enriched in MKI67⁺ tumor cells and essential for β1‑integrin glycosylation, FAK–PI3K–AKT activation and cell adhesion. Its overexpression also upregulates MMP2 through FAK, disrupts the blood–retinal barrier and promotes invasion. Importantly, structure‑based screening identifies myricoside, which inhibits B4GALT3 and mimics knockdown effects in vitro and in an orthotopic model. These findings identify B4GALT3 as a promising therapeutic target for advanced RB.

Aberrant B4GALT3 expression has been reported in various cancers, but its role remains controversial [14–16, 43, 44]. Consistent with our findings in RB, B4GALT3 is highly expressed and correlates positively with tumor progression in neuroblastoma by enhancing β1‑integrin glycosylation, stabilizing its activity, and promoting invasion [14]. In contrast, studies in colorectal cancers suggest that B4GALT3 may instead suppress cell–ECM adhesion independently of β1‑integrin, possibly through broader modification of other glycoprotein or glycolipid targets [16]. These observations highlight the complexity of B4GALT3’s function and its distinct roles across tumor types. The B4GALT3 gene is located at chromosome 1q23.3, and amplification of 1q is a recurrent somatic copy number alteration (SCNA) in RB. Numerous studies have shown that SCNAs in RB are closely associated with tumor biological behavior; for example, gains of 1q and 6p and loss of 16q have been linked to increased local invasion [45]. Therefore, B4GALT3 may represent a key gene within the amplified 1q region that contributes to enhanced RB invasiveness. At the molecular level, previous studies have identified poly-N-acetyllactosamine structures on the β1 subunit of α3β1 integrin, a receptor for laminin, fibronectin, and collagen [46, 47]. B4GALT3 has been shown to catalyze the formation of these poly-N-acetyllactosamine chains [48]. Using RCA-I lectin binding, which specifically recognizes galactose-containing structures such as poly-N-acetyllactosamine, we found that modulation of B4GALT3 expression alters the level of poly-N-acetyllactosamine on β1 integrin in RB cells. Notably, B4GALT3 is also a key enzyme in the keratan sulfate (KS) biosynthesis pathway. The KS proteoglycan lumican—a small leucine‑rich proteoglycan—has been reported to influence ECM organization and to bind integrins, thereby affecting tumor progression [49, 50]. We found that lumican is broadly expressed in RB and that B4GALT3 knockdown reduces lumican levels (Fig. S3), suggesting that B4GALT3 may regulate integrin function through multiple glycosylation‑dependent mechanisms.

Functionally, our study found that B4GALT3 overexpression enhances β1 integrin glycosylation in RB cells, activating the FAK–PI3K–AKT signaling pathway and thereby promoting cell proliferation and adhesion to fibronectin (FN). This is consistent with the unique adhesion profile of RB suspension cells such as Y79, which lack integrin α subunits (e.g., α1, α2, α3) required for laminin or type IV collagen binding but retain α4β1 integrin-mediated FN adhesion [51]. Clinically, optic nerve invasion by RB—particularly beyond the lamina cribrosa—is strongly associated with an increased risk of brain metastasis [52, 53]. The optic nerve microenvironment is enriched with extracellular matrix components, including FN and laminin, as demonstrated in pathological mouse models [54]. The B4GALT3-mediated enhancement of FN adhesion may provide RB cells with a context-dependent advantage, facilitating directed migration along the optic nerve through strengthened integrin-mediated adhesion combined with increased proliferation.

RB can present as either endophytic or exophytic tumors, each with distinct growth patterns that shape their invasive potential [55]. Endophytic tumors grow predominantly into the vitreous, whereas exophytic tumors originate in the outer retina and expand into the subretinal space [56]. This outward growth necessitates breaching the outer blood–retina barrier, which includes the choroid, Bruch’s membrane, and the retinal pigment epithelium [57]. A pivotal mechanism driving this barrier disruption is the degradation of the extracellular matrix (ECM) by matrix metalloproteinases (MMPs) [58]. Among them, MMP2 is well established as a critical mediator of tumor invasion across cancers, including RB, due to its ability to degrade type IV collagen, a major component of basement membranes [59–61]. Using a co-culture system of RB and ARPE-19 cells, adapted from an established in vitro triple-culture model for preclinical RB research [62], our study demonstrates that B4GALT3-mediated glycosylation of β1 integrin activates FAK signaling, which upregulates MMP2 and promotes ECM degradation. This provides new insight into how exophytic tumors overcome anatomical barriers.

Biofluids such as plasma and aqueous humor are increasingly being investigated for the detection of circulating tumor DNA (ctDNA) or cell-free DNA (cfDNA) in RB, offering a minimally invasive approach to inform treatment decisions, monitor therapeutic responses, and provide prognostic guidance [63–66]. Notably, studies in other cancer types have highlighted the utility of B4GALT family members as circulating biomarkers. For example, B4GALT1 hypermethylation has been reported as a predictive cfDNA biomarker for cetuximab response in colorectal cancer [67], while B4GALT2 has been identified in extracellular vesicles as a promising diagnostic biomarker for hepatocellular carcinoma [68]. These findings suggest that B4GALT3, given its overexpression and functional relevance in RB, may similarly hold potential as a liquid biopsy biomarker, enabling non-invasive tumor monitoring.

In this study, we identified myricoside—a natural compound isolated from the aerial parts of *Phlomis oppositiflora* with limited prior investigation in cancer [69]—as a potential inhibitor of B4GALT3. Molecular docking and biochemical assays suggest that myricoside directly interacts with the B4GALT3 protein to suppress its expression. Strikingly, treatment with myricoside significantly reduced RB tumor growth, impaired cell adhesion, and induced tumor cell apoptosis, underscoring B4GALT3 as a promising therapeutic target for limiting local invasion in RB. Beyond myricoside, virtual screening and in vitro validation also highlighted Plantainoside D as another promising candidate. Although primarily reported for anti-inflammatory and cardioprotective functions through pathways such as ROS/NF-κB, VDCC/PKC, and Sirt3/NLRP3 [70–72], Plantainoside D exhibited notable inhibitory activity in RB cells and ranked among the top compounds in the virtual screen. These findings suggest that Plantainoside D may also possess anti-RB potential and warrant further investigation.

In conclusion, our findings reveal the critical role of B4GALT3 in regulating β1-integrin glycosylation and FAK-mediated signaling in RB, and provide insights into the mechanism of B4GALT3-driven tumor proliferation, adhesion, and invasion. These findings not only disclose the link between aberrant glycosylation and RB malignancy but also highlight the promising strategy of targeting B4GALT3 to improve eye-preserving outcomes in advanced RB.

## Supplementary information

Supplementary Information

## Data Availability

The raw sequence data reported in this paper have been deposited in the Genome Sequence Archive in the National Genomics Data Center [73, 74], China National Center for Bioinformation/Beijing Institute of Genomics, Chinese Academy of Sciences (GSA-Human: HRA012394) that are publicly accessible at https://ngdc.cncb.ac.cn/gsa-human. All data supporting the current study are provided in the article, Supplementary Information.
