# Supplementary material for: β1,4-galactosyltransferase III drives retinoblastoma invasion via activation of integrin-FAK axis
Source: Cell Death Dis. 2026 Mar 23;17(1):336. doi: 10.1038/s41419-026-08620-5 (PMC13039461; doi:10.1038/s41419-026-08620-5)
Supplement: Supplementary file 1 — Supplementary Information [file 41419_2026_8620_MOESM1_ESM.pdf]

Supplementary Information

Supplemental Method Statement: Details of experimental design of animal studies

For the orthotopic xenograft experiments, the following details of experimental design and statistical considerations were implemented, which complement the main text description:

Sample size determination: The sample size (n = 8 mice per group) was determined based on prior studies with similar orthotopic xenograft models[1], which reported that the similar sample size was sufficient to detect biologically meaningful differences in tumor growth and therapeutic responses consistent with the endpoints evaluated in the current study.

Inclusion and exclusion criteria: Inclusion criteria were predefined as female BALB/c nude mice aged 4–6 weeks, weighing 18–20 g, with no signs of ocular abnormalities or systemic disease. Exclusion criteria included unexpected death within 72 h post-injection, failure of cell injection, or absence of detectable tumor formation by day 10 post-injection. No mice were excluded in this study.

Randomization: Mice were randomly assigned to experimental groups (wild-type, B4GALT3 knockdown, B4GALT3 overexpression, myricoside-treated, and vehicle-treated) prior to cell injection. The randomization was performed by an investigator (M.W.) not involved in subsequent outcome assessment.

Blinding: Outcome assessment (tumor formation evaluation at day 30 post-injection, histological analysis, and protein quantification) was conducted by two independent investigators (J.L., Y.L.) blinded to group allocation.

1. Tang J, Liu Y, Wang Y, Zhang Z, Nie J, Wang X*, et al.* Deciphering metabolic heterogeneity in retinoblastoma unravels the role of monocarboxylate transporter 1 in tumor progression. *Biomarker research* 2024, **12**(1)**:** 48.

Table S1. The primer sequences for qPCR

Gene	Sequences
B4GALT1	5'-CTATATCTCGCCCAAATGCTG-3' (forward)
B4GALT1	5'-GTGCAATTCGGTCAAACCTC-3' (reverse)
B4GALT2	5'-GGGCAGACTGCTGATCGAG-3' (forward)
B4GALT2	5'-CCGGTGTCTAAAGGGGATGAT-3' (reverse)
B4GALT3	5'-CGAGATCAGGGACCGACATTT-3' (forward)
B4GALT3	5'-GATCGTTCTGGACAGTAGGGC-3' (reverse)
B4GALT4	5'-CATCCCAAGCATCTGGTGGT-3' (forward)
B4GALT4	5'-TCCCCATCCCCAGTAGTTGT-3' (reverse)
GAPDH	5'-GGAGCGAGATCCCTCCAAAAT-3' (forward)
GAPDH	5'-GGCTGTTGTCATACTTCTCATGG-3' (reverse)

Table S2. Antibody Information

Antibody	Catalog No.	Company	RRID	Dilution
B4GALT3	11041-1-AP	Proteintech	AB_2290072	WB: 1:1000; IF: 1:100
GAPDH	10494-1-AP	Proteintech	AB_2263076	WB: 1:5000
Lumican	ab168348	Abcam	AB_2920864	WB: 1:1000; IF: 1:100
Integrin β1	26918-1-AP	Proteintech	AB_2880685	WB: 1:1000; IF: 1:100
T-FAK	3285	Cell Signaling Technology	AB_2269034	WB: 1:1000
P-FAK	381143	ZEN-BIO	AB_3271521	WB: 1:500
P-FAK	83933-1-RR	Proteintech	AB_3671514	IF: 1:200
T-PI3K	4257	Cell Signaling Technology	AB_659889	WB: 1:1000
P-PI3K	341468	ZEN-BIO	AB_3675929	WB: 1:500
T-AKT	342529	ZEN-BIO	AB_3675962	WB: 1:500
P-AKT	310021	ZEN-BIO	AB_3675963	WB: 1:500
BCL2	381702	ZEN-BIO	AB_2924433	WB: 1:500
MMP2	380817	ZEN-BIO	AB_2917993	WB: 1:500; IF: 1:100
ZO-1	21773-1-AP	Proteintech	AB_10733242	WB: 1:2000; IF: 1:500
Occludin	91131	Cell Signaling Technology	AB_2934013	WB: 1:1000; IF: 1:200
Ki67	GB111499	Servicebio	AB_2927572	IF: 1:200

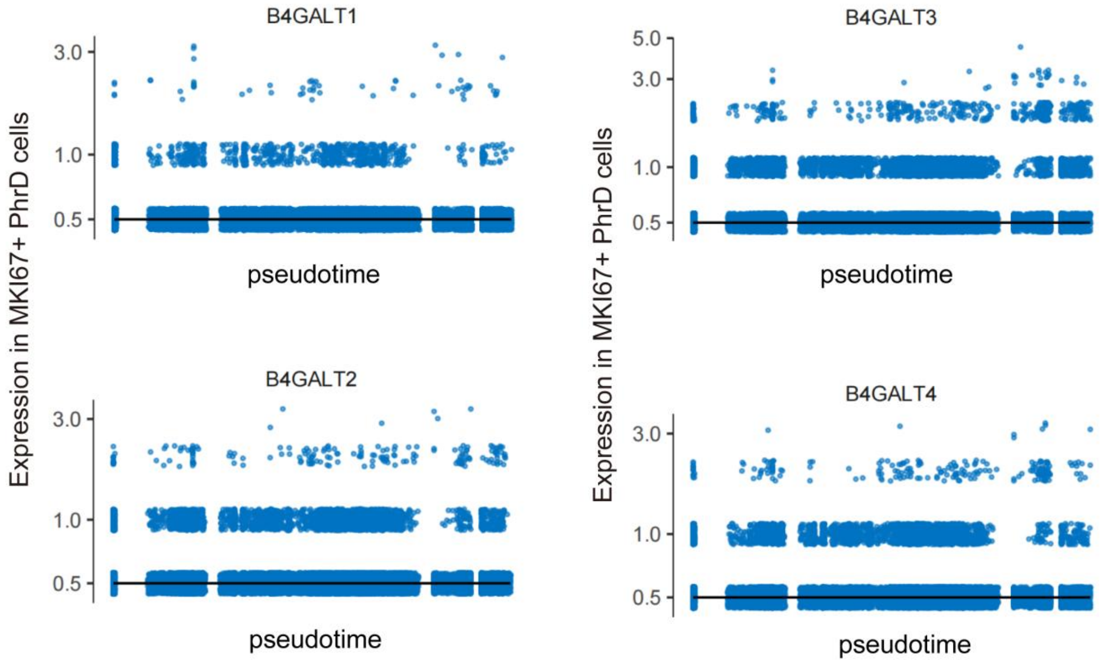

Figure S1. Pseudotemporal dynamics of *B4GALT* family gene expression in MKI67⁺ PhrD cells. *B4GALT1* and *B4GALT2* exhibit low and stable expression, while *B4GALT3* and *B4GALT4* show upregulated expression and increased dispersibility during this progression.

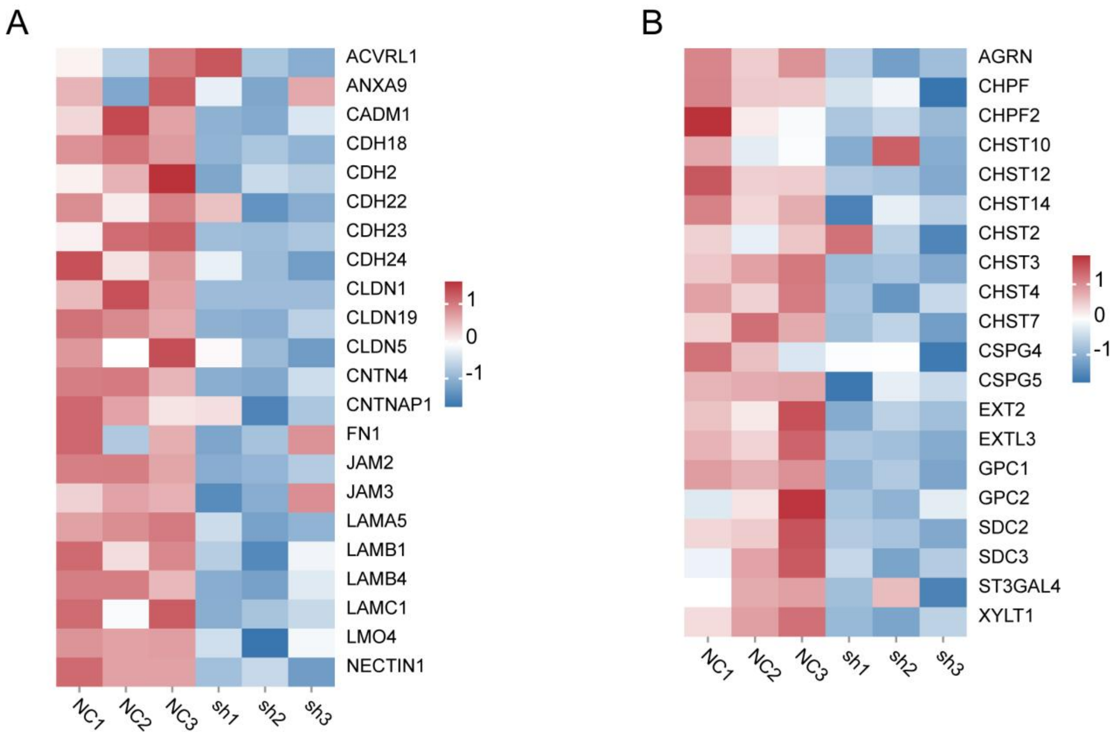

Figure S2. Heatmaps showing differentially expressed genes between control (NC) and shB4GALT3 groups from RNA-seq analysis. (A) Expression profiles of genes enriched in cell adhesion－related pathways. (B) Expression patterns of genes involved in the glycosaminoglycan biosynthesis pathway.

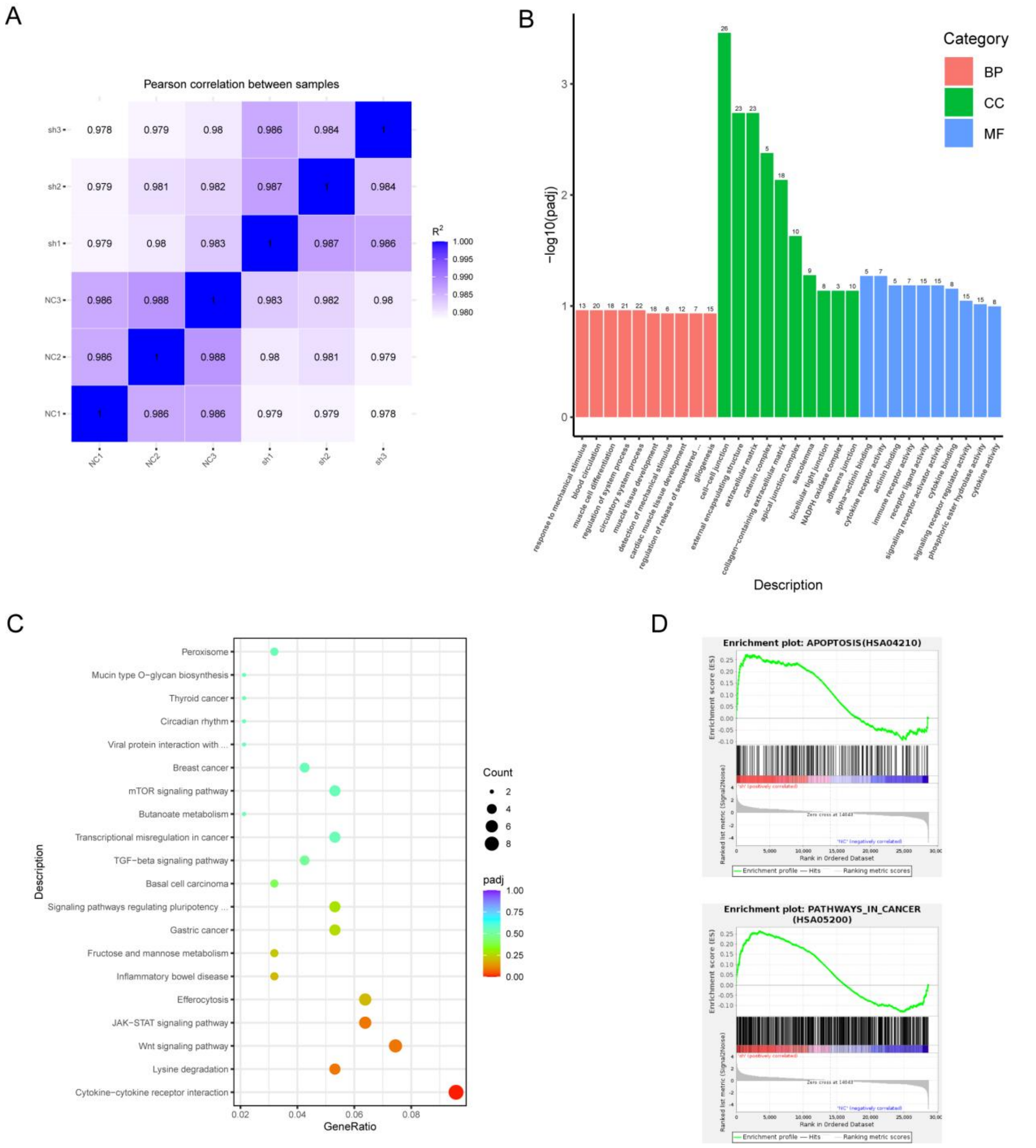

Figure S3. Gene expression profiling of B4GALT3 knockdown in WERI-Rb1 cells. **A.** Pearson correlation analysis of RNA-seq data between shNC and shB4GALT3 in WERI-Rb1 cells. **B.** Gene Ontology (GO) analysis of differentially expressed genes (DEGs) between shNC and shB4GALT3 in WERI-Rb1 cells, including biological process (BP), cellular component (CC), and molecular function (MF). **C.** KEGG pathway enrichment analysis of upregulated DEGs in shB4GALT3-treated WERI-Rb1 cells. **D.** Gene Set Enrichment Analysis (GSEA) showing enrichment of apoptosis and cancer-related pathways in shB4GALT3-treated WERI-Rb1 cells.

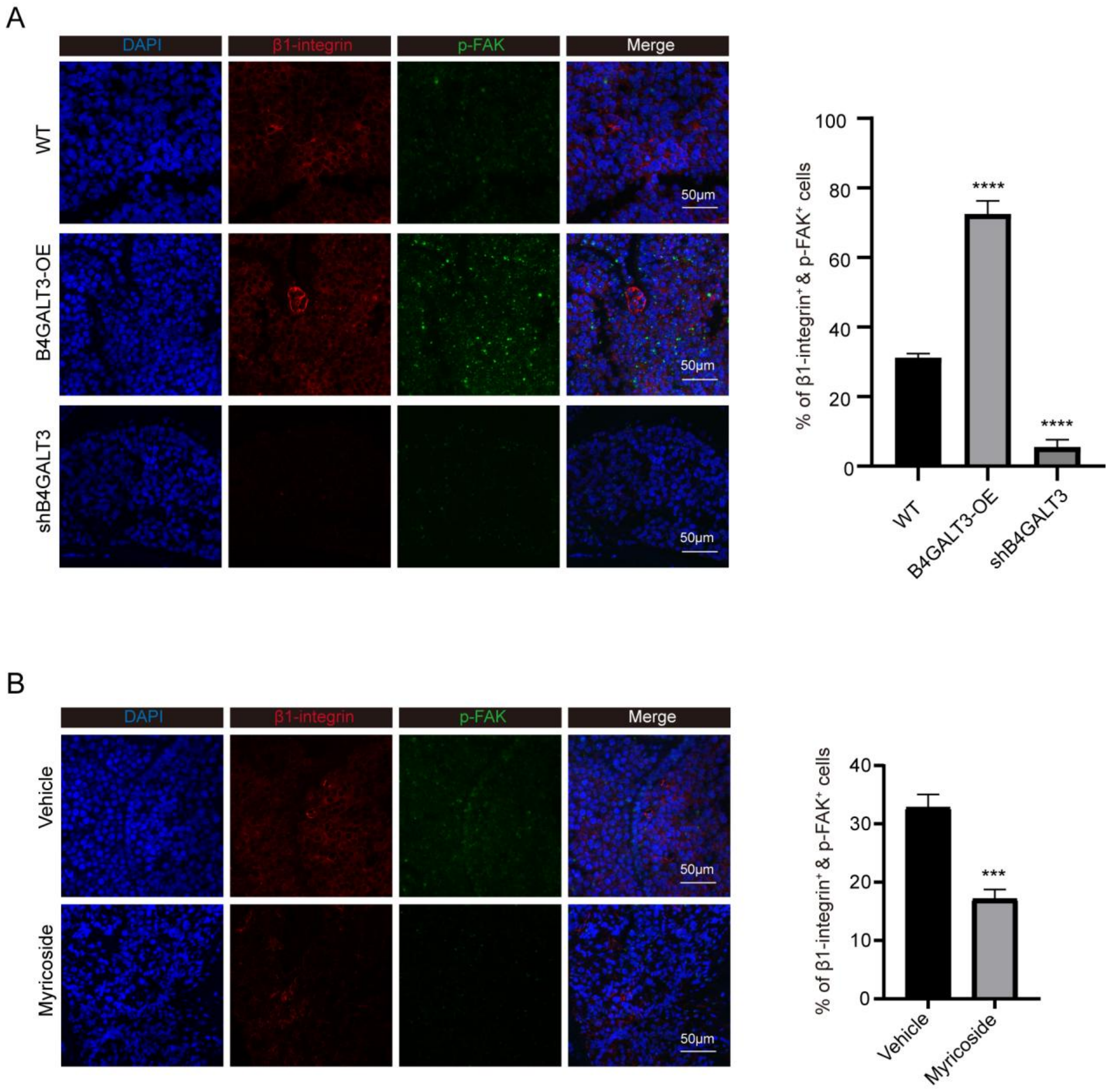

Figure S4. Colocalization of ITGB1 and p-FAK in tumor tissues. (A) Representative immunofluorescence images showing colocalization of ITGB1 and p-FAK in tumor sections from WT, B4GALT3-overexpressing (OE), and B4GALT3-knockdown (sh) groups. Quantitative analysis of $ITGB1^{+}$ & $p\text{-}FAK^{+}$ cells was performed using one-way ANOVA. $^{****}P < 0.0001$. (B) Representative images and quantification of ITGB1 and p-FAK colocalization in tumor tissues from vehicle-treated and myricoside-treated groups. Statistical analysis was performed using an unpaired t-test. $^{***}P<0.001$.

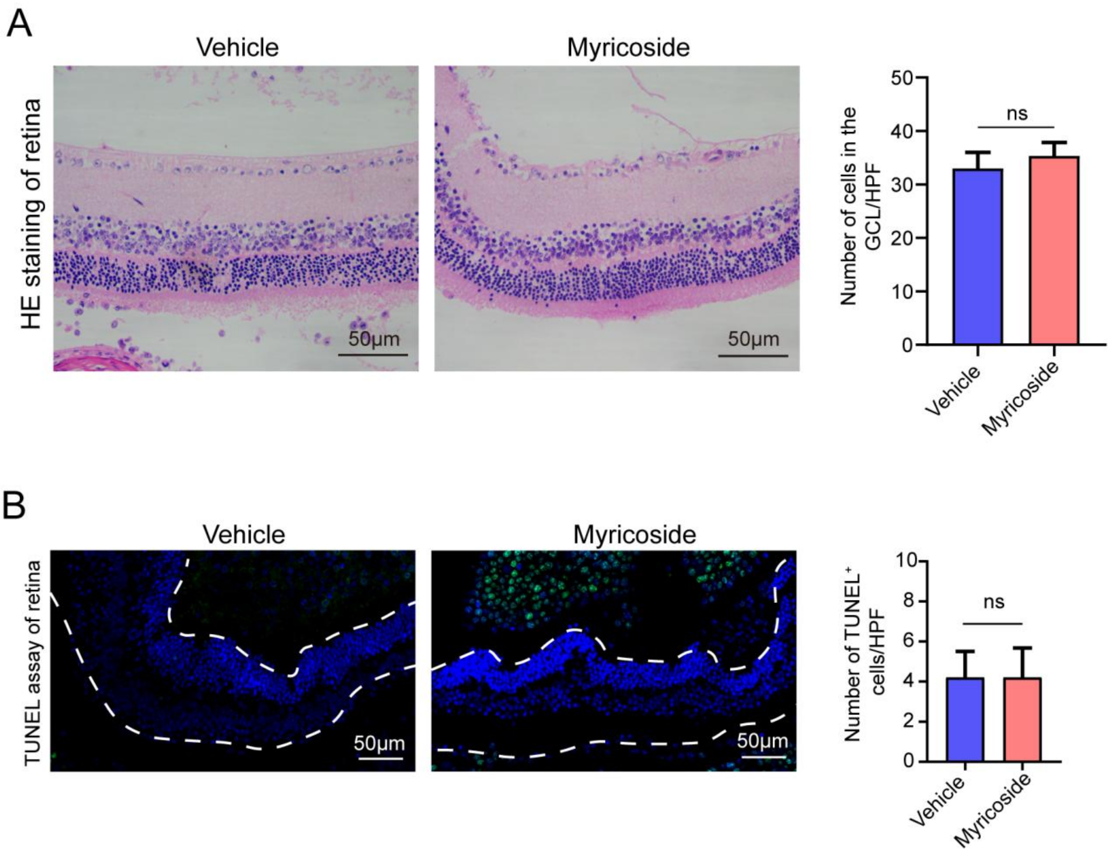

Figure S5. Evaluation of retinal safety following intraocular administration of Myricoside. (A) Representative H&E–stained retinal sections from vehicle- and myricoside-treated eyes. Quantification of the cell number in the ganglion cell layer (GCL) per high-power field (HPF) was performed using an unpaired t-test. ns, not significant. (B) Representative TUNEL-stained retinal sections showing apoptotic cells in vehicle- and myricoside-treated groups. Quantification of TUNEL-positive cells per field was performed using an unpaired t-test. ns, not significant.

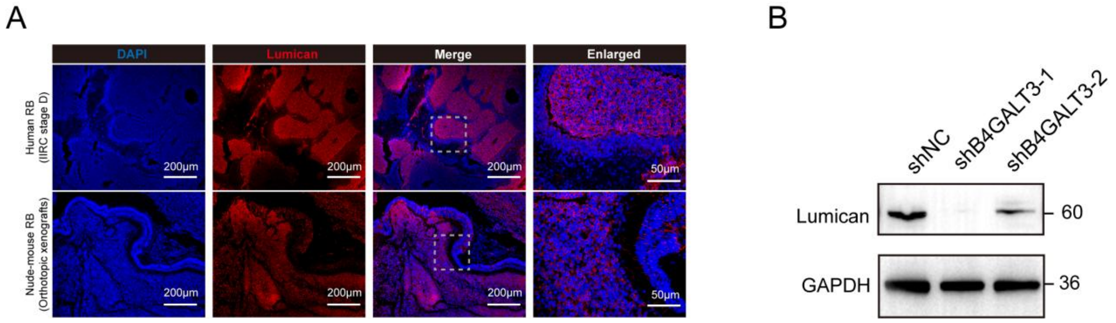

Figure S6. B4GALT3 modulates Lumican expression in retinoblastoma. A. Representative immunofluorescence (IF) staining images of Lumican in IIRC stage D human retinoblastoma (RB) and nude-mouse RB xenograft tissues. **B.** Western blot analysis of Lumican expression in Y79 cells following B4GALT3 knockdown.

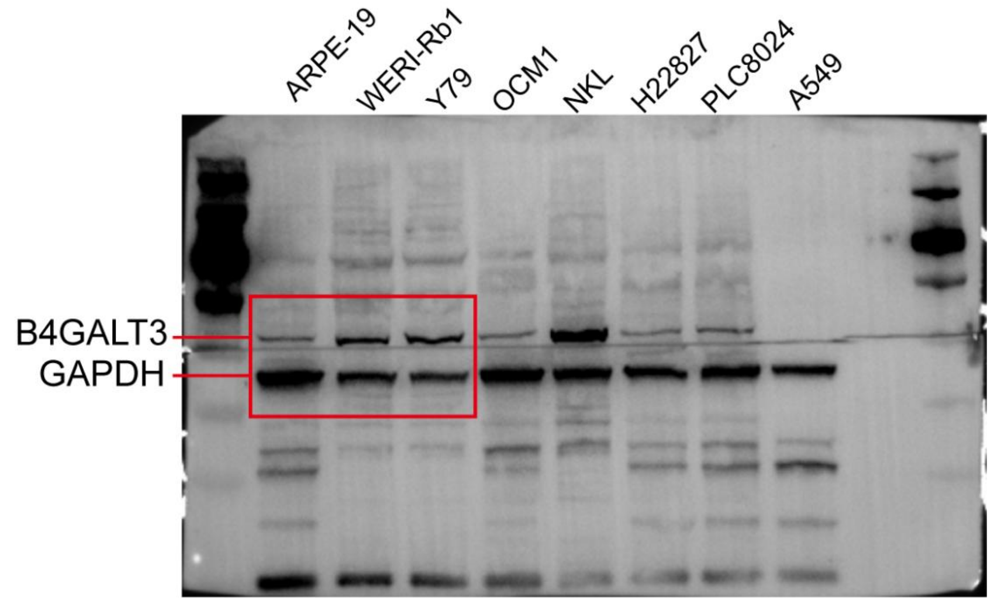

Figure S7. Uncropped original western blots related to Figure 1 H.

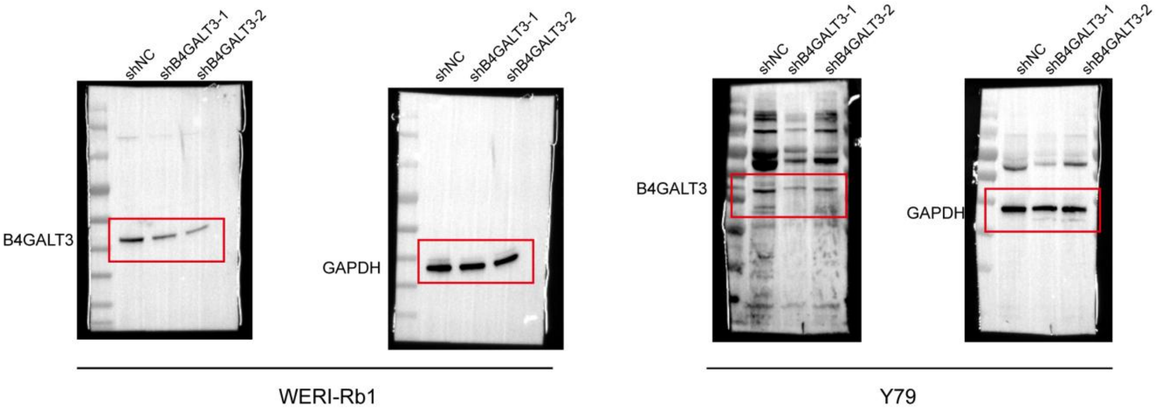

Figure S8. Uncropped original western blots related to Figure 2 A.

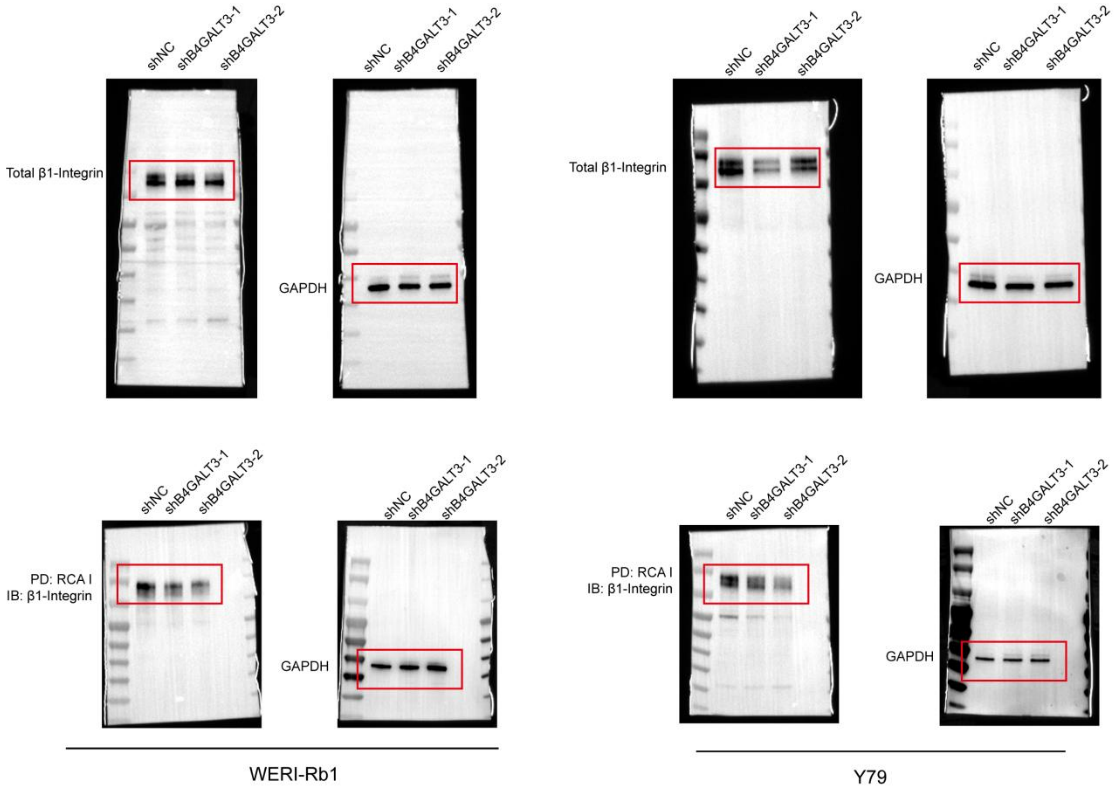

Figure S9. Uncropped original western blots related to Figure 2 H, I.

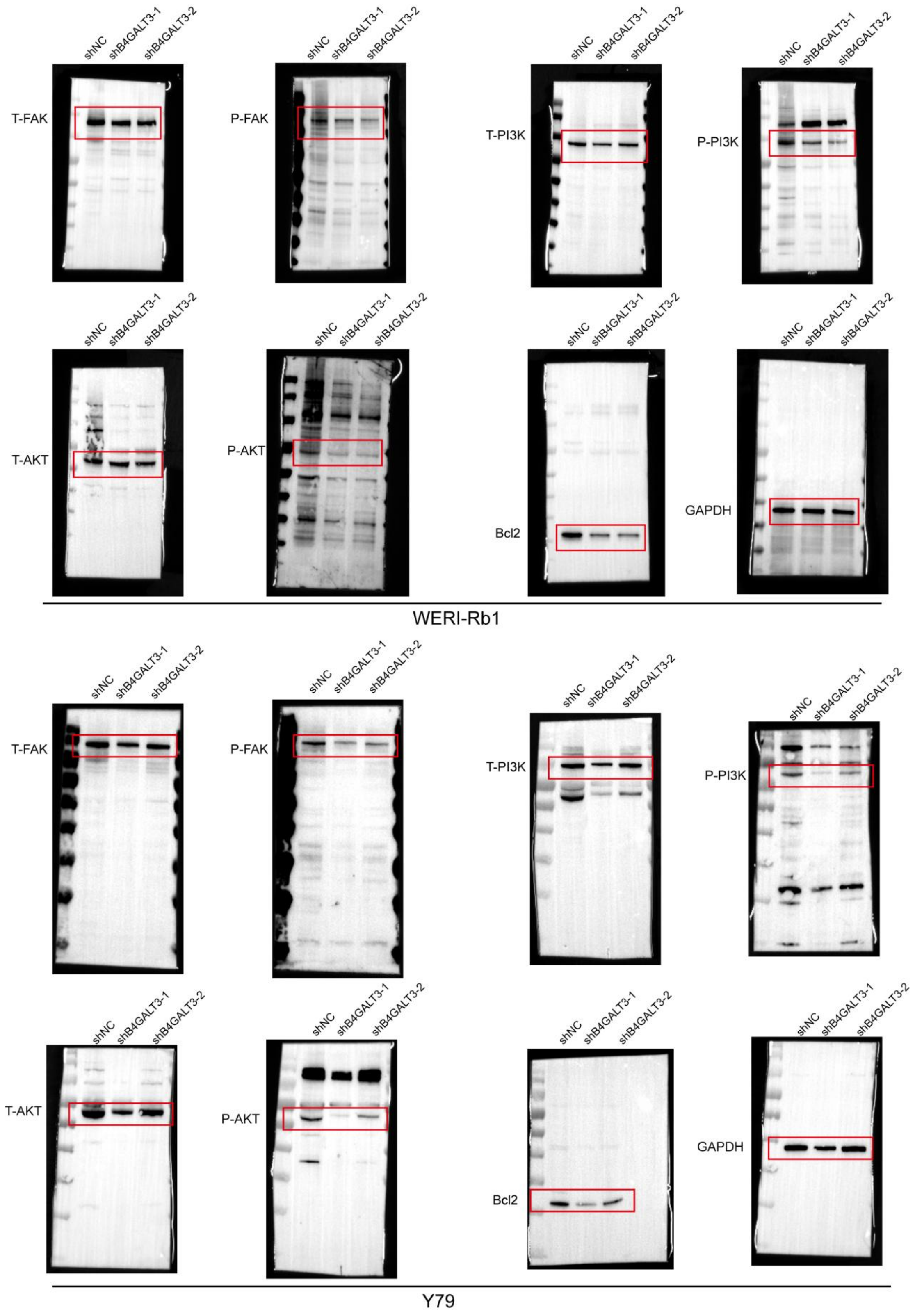

Figure S10. Uncropped original western blots related to Figure 2 J, K.

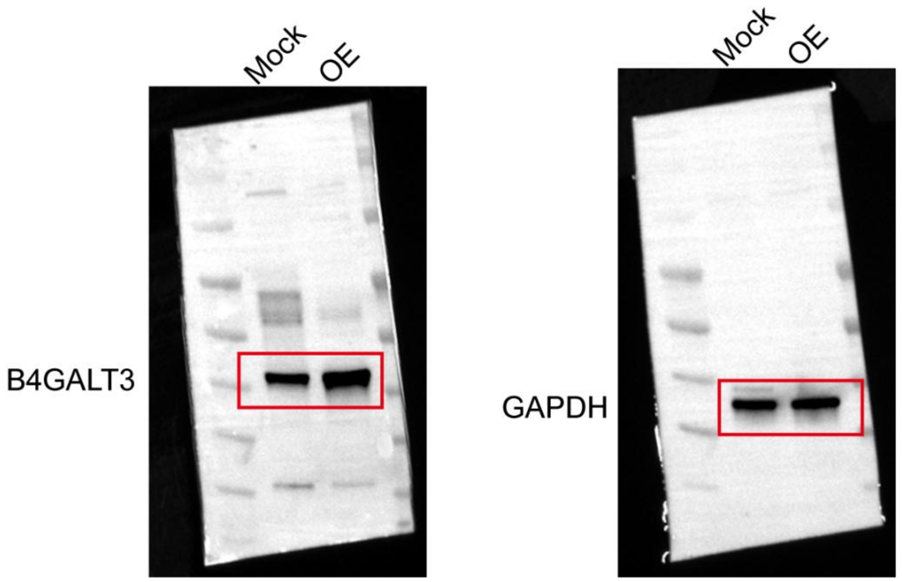

Figure S11. Uncropped original western blots related to Figure 3 A.

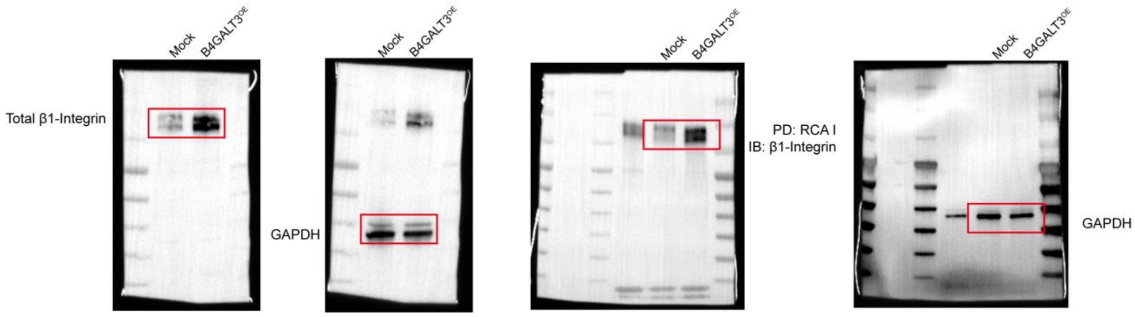

Figure S12. Uncropped original western blots related to Figure 3 E.

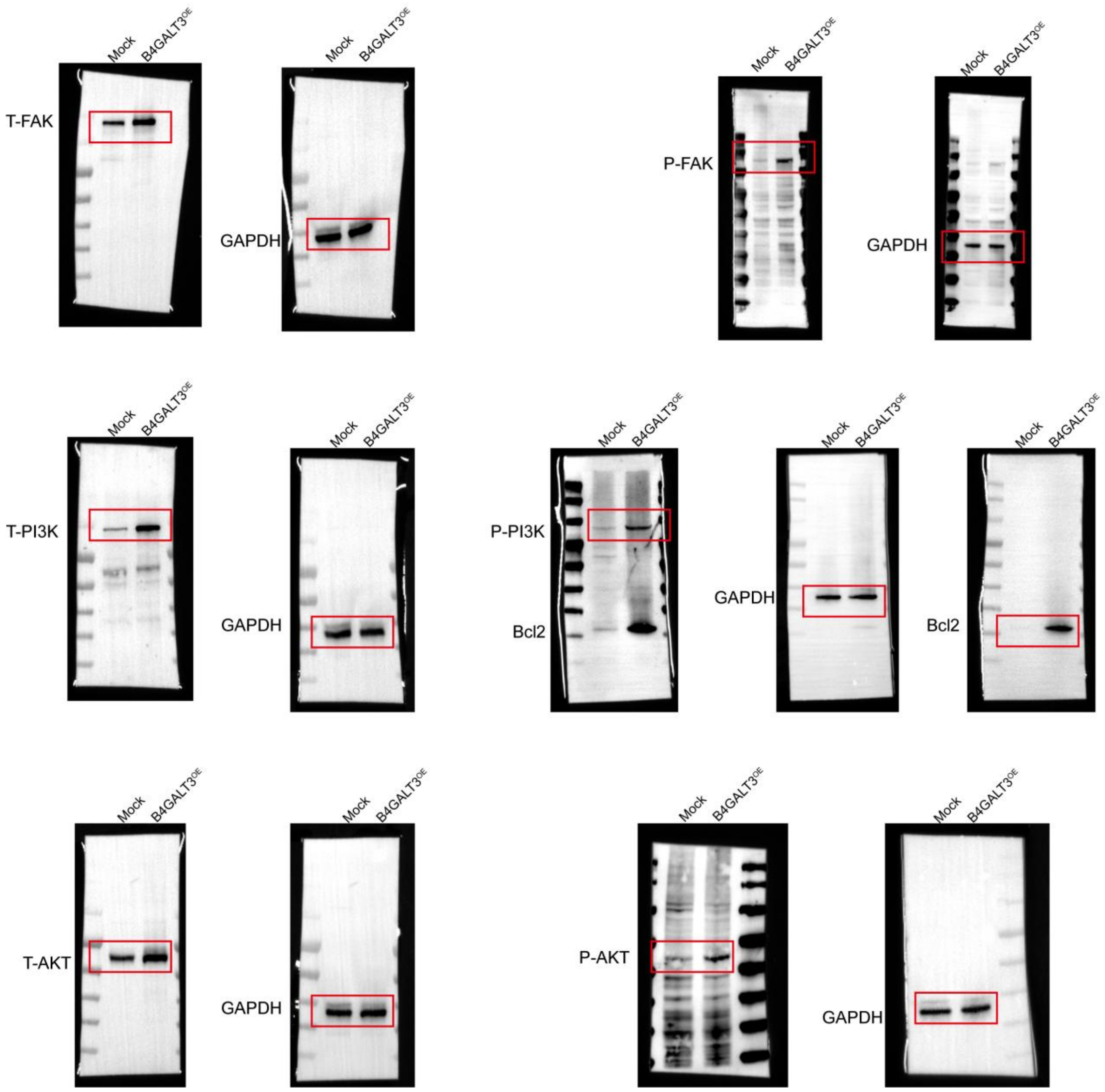

Figure S13. Uncropped original western blots related to Figure 3 F.

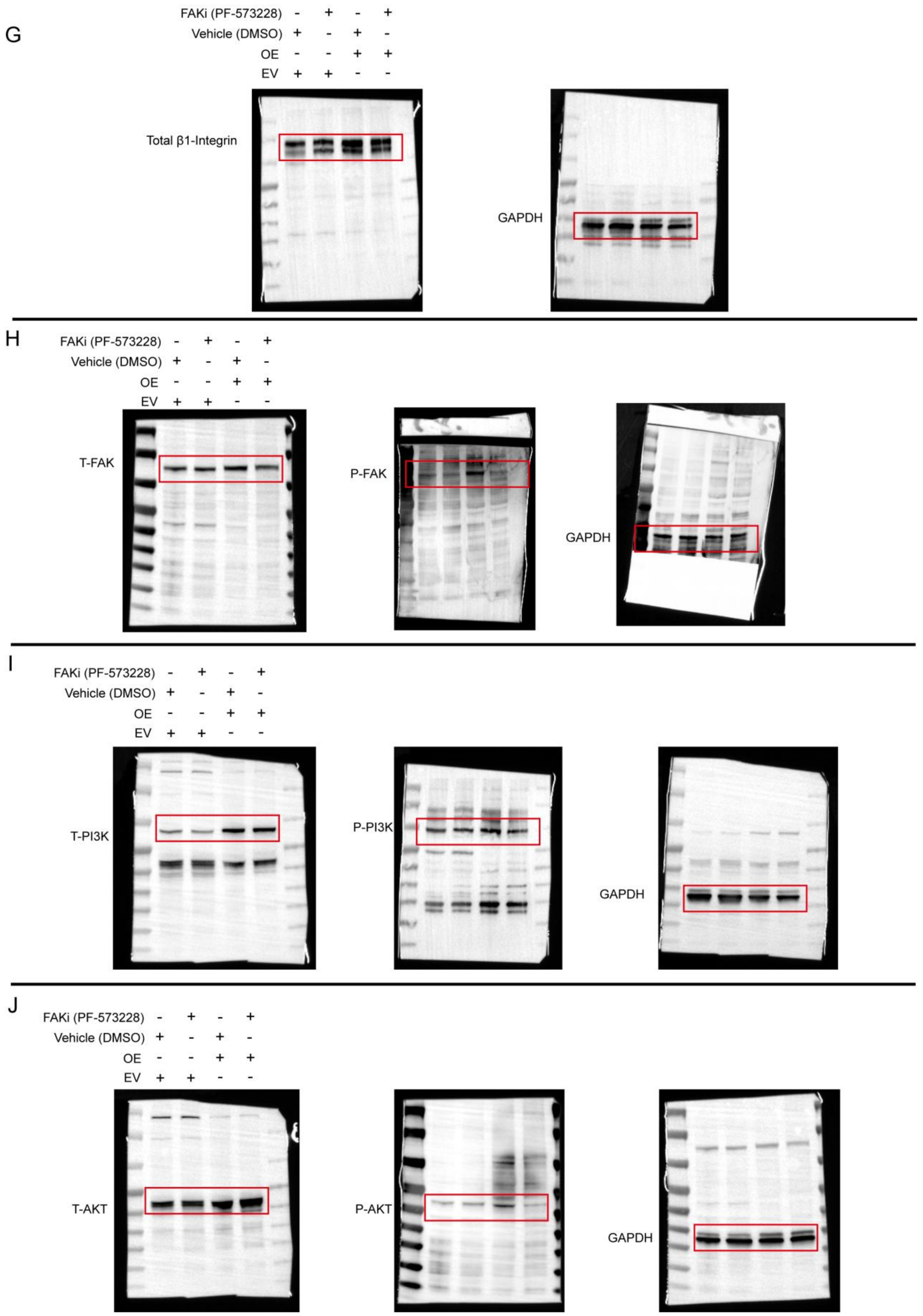

Figure S14. Uncropped original western blots related to Figure 3 G, H, I, J.

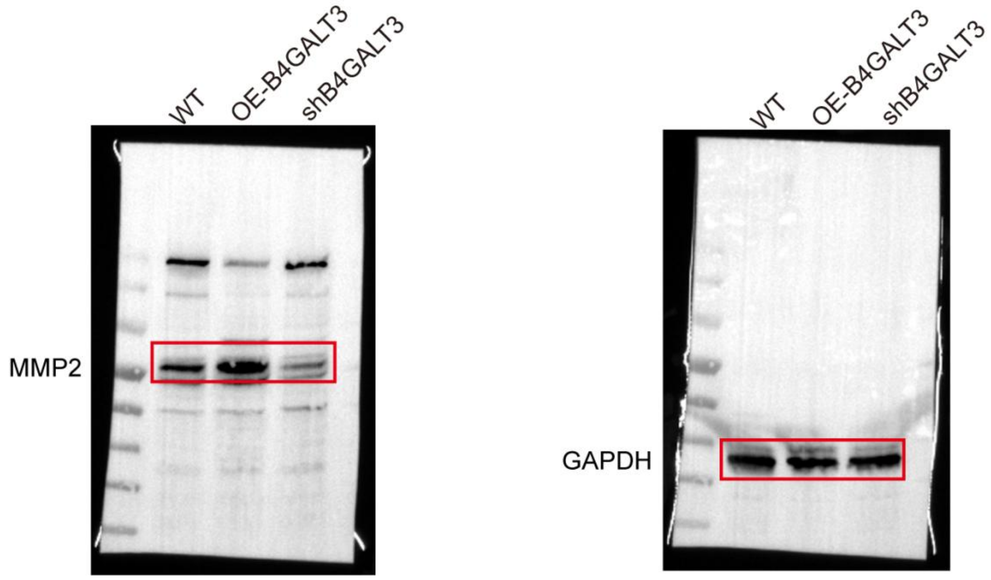

Figure S15. Uncropped original western blots related to Figure 4 B.

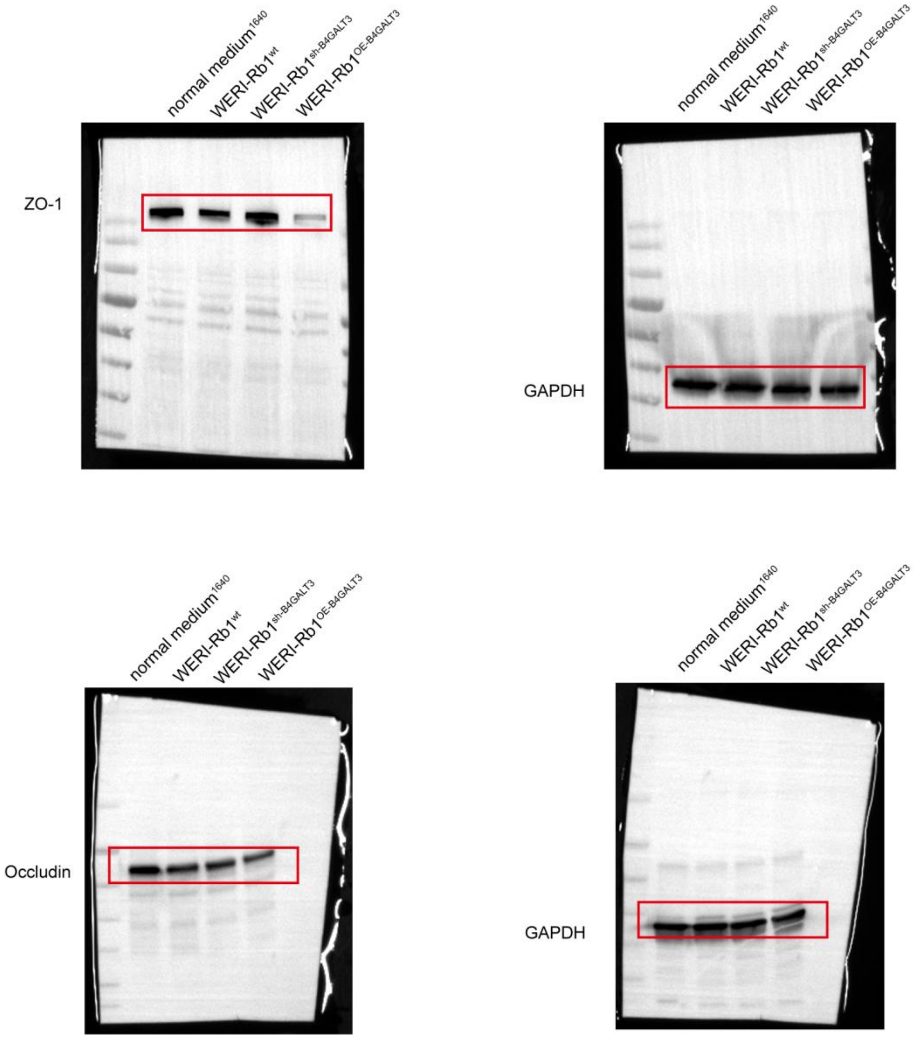

Figure S16. Uncropped original western blots related to Figure 4 F.

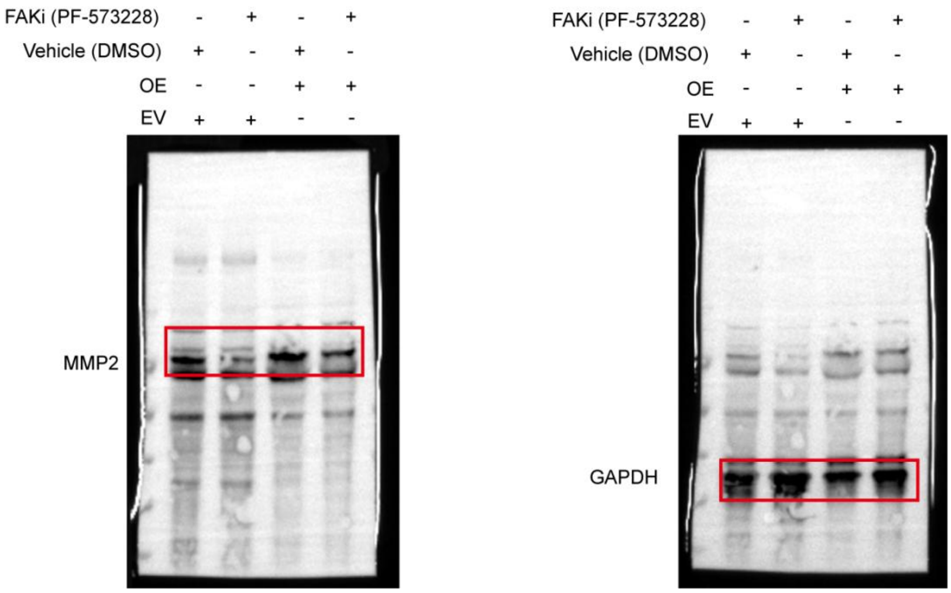

Figure S17. Uncropped original western blots related to Figure 4 H.

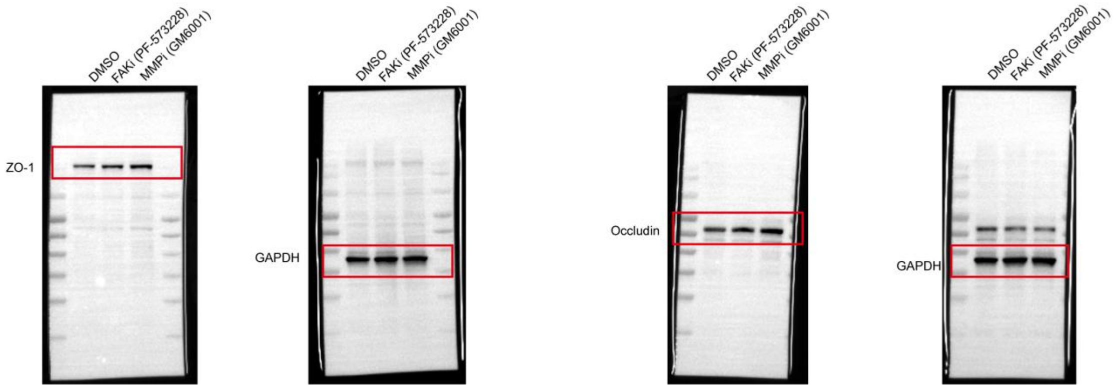

Figure S18. Uncropped original western blots related to Figure 4 J.

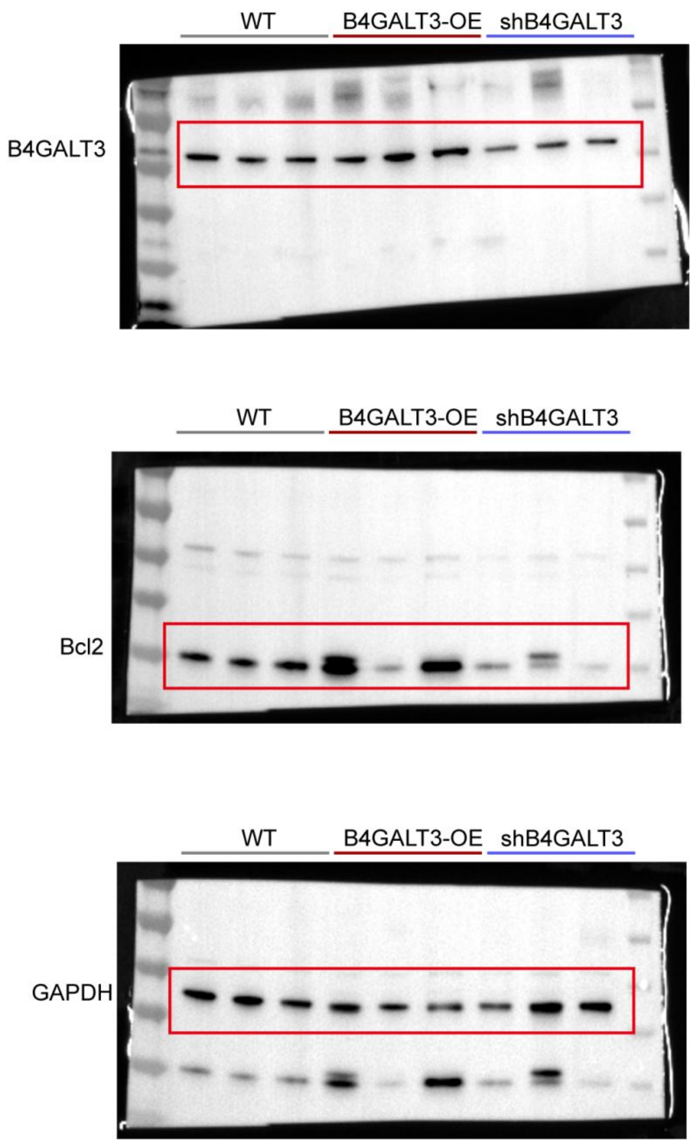

Figure S19. Uncropped original western blots related to Figure 5 L.

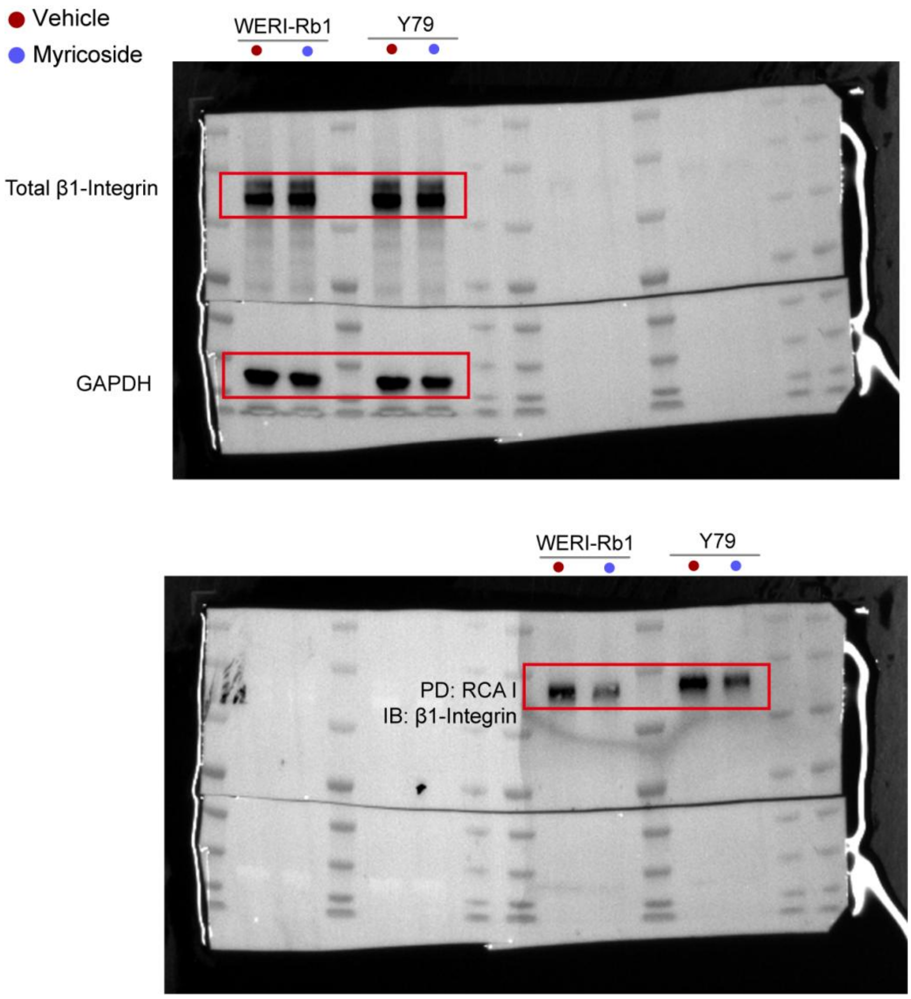

Figure S20. Uncropped original western blots related to Figure 6 I.

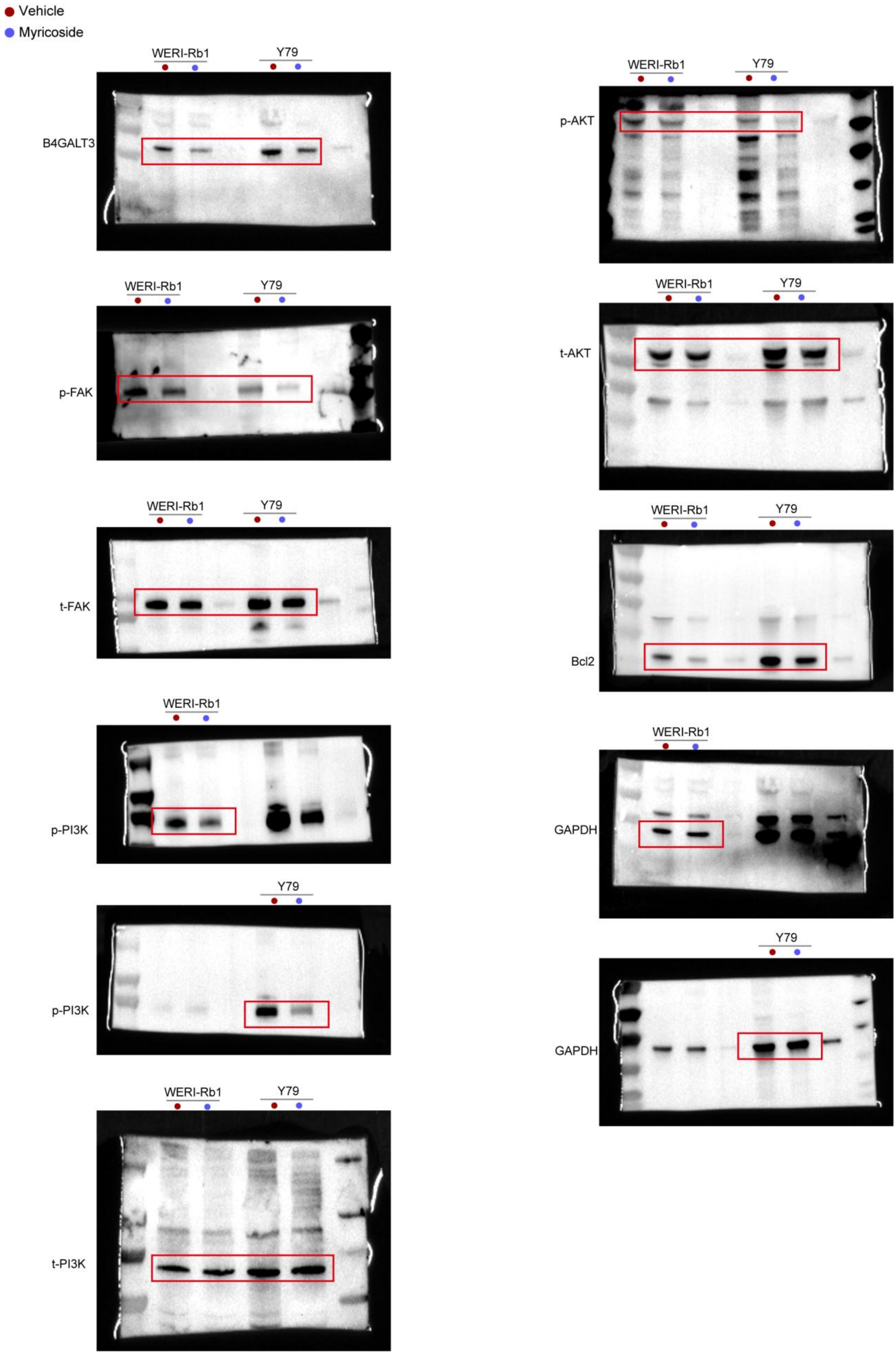

Figure S21. Uncropped original western blots related to Figure 6 J.

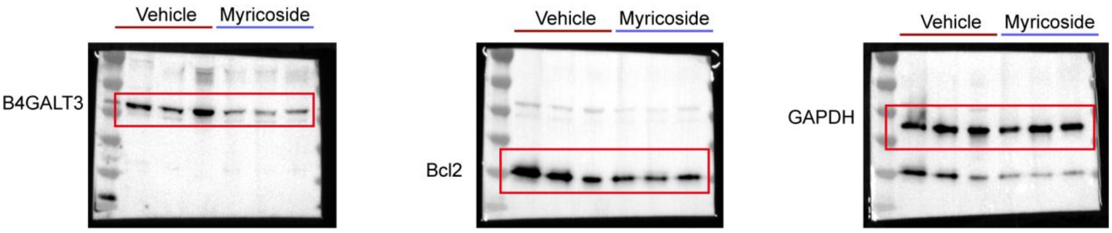

Figure S22. Uncropped original western blots related to Figure 7 L.
